# Single‐Component Electroactive Polymer Architectures for Non‐Enzymatic Glucose Sensing

**DOI:** 10.1002/advs.202308281

**Published:** 2024-03-23

**Authors:** Christina J. Kousseff, Shofarul Wustoni, Raphaela K. S. Silva, Ariel Lifer, Achilleas Savva, Gitti L. Frey, Sahika Inal, Christian B. Nielsen

**Affiliations:** ^1^ Department of Chemistry Queen Mary University of London Mile End Road London E1 4NS UK; ^2^ Organic Bioelectronics Laboratory Biological and Environmental Science and Engineering King Abdullah University of Science and Technology (KAUST) Thuwal 23955‐6900 Saudi Arabia; ^3^ Department of Materials Science and Engineering Technion–Israel Institute of Technology Haifa 32000 Israel; ^4^ Bioelectronics Section Department of Microelectronics Faculty of Electrical Engineering, Mathematics and Computer Science (EEMCS) Delft University of Technology Delft 2628 CD The Netherlands

**Keywords:** electropolymerization, glucose sensor, organic bioelectronics, organic electrochemical transistors, PEDOT

## Abstract

Organic mixed ionic‐electronic conductors (OMIECs) have emerged as promising materials for biological sensing, owing to their electrochemical activity, stability in an aqueous environment, and biocompatibility. Yet, OMIEC‐based sensors rely predominantly on the use of composite matrices to enable stimuli‐responsive functionality, which can exhibit issues with intercomponent interfacing. In this study, an approach is presented for non‐enzymatic glucose detection by harnessing a newly synthesized functionalized monomer, **EDOT‐PBA**. This monomer integrates electrically conducting and receptor moieties within a single organic component, obviating the need for complex composite preparation. By engineering the conditions for electrodeposition, two distinct polymer film architectures are developed: pristine **PEDOT‐PBA** and molecularly imprinted **PEDOT‐PBA**. Both architectures demonstrated proficient glucose binding and signal transduction capabilities. Notably, the molecularly imprinted polymer (MIP) architecture demonstrated faster stabilization upon glucose uptake while it also enabled a lower limit of detection, lower standard deviation, and a broader linear range in the sensor output signal compared to its non‐imprinted counterpart. This material design not only provides a robust and efficient platform for glucose detection but also offers a blueprint for developing selective sensors for a diverse array of target molecules, by tuning the receptor units correspondingly.

## Graphical abstract

A synthetically tailored electroactive monomer with binding affinity for sugars is used to fabricate 3D polymer scaffolds via electrodeposition. By controlling the polymer deposition conditions, mixed ionic‐electronic conducting polymer films with built‐in selectivity toward glucose are obtained without the use of enzymes or other external mediators. Proof‐of‐concept organic electrochemical transistor‐based sensors are fabricated to evaluate the sensor performance.

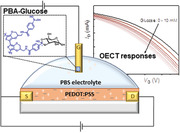

## 1 Introduction

Glucose, a quintessential hexose sugar, is a ubiquitous constituent of the human diet and serves as the major precursor of the metabolic pathway, providing energy for higher respiring organisms.^[^ ^1^ ^]^ The prominence of glucose in the human body, along with its critical role in metabolism, renders it a compelling biomarker of interest for biological sensing. In humans, its normal concentration in the bloodstream ranges from 4 to 7 mm.^[^ ^2^ ^]^ Dysregulation of glucose levels can lead to significant health complications, such as hyperglycaemia^[^ ^3^ ^]^ and hypoglycaemia,^[^ ^4^ ^]^ as well as diabetes mellitus. Notably, diabetes represents one of the most prevalent diseases worldwide, which affected 529 million people worldwide in 2021, a figure projected to rise to 1.31 billion by 2050.^[^ ^5^ ^]^ As of now, there is no known cure, and its management necessitates the continuous monitoring of glucose concentrations in the bloodstream.

Existing commercial methods for monitoring glucose levels in diabetes patients, and alternatives found in the scientific literature, predominantly rely on the use of the glucose oxidase (GOx) enzyme, due to its high specificity and its capacity as a redox‐active molecule that facilitates straightforward conversion to an electronic signal. Various organic and inorganic conducting materials have been integrated with GOx to detect glucose through different transduction mechanisms. Organic mixed ionic‐electronic conductors (OMIECs) have emerged as a transducing material promising for chemical sensing, owing to their electrochemical functionality,^[^ ^6^ ^]^ stability in an aqueous environment,^[^ ^7^ ^]^ and biocompatibility.^[^ ^8^ ^]^ The ability to modify OMIECs at both molecular and macroscopic levels enables a high degree of control over structure‐function relationships. For instance, polar side chains such as oligoethers can be introduced to facilitate the diffusion of hydrated ions into the bulk material, enhancing the interface with biological systems,^[^ ^9^, ^10^ ^]^ while tailored binding groups can interact with biological analytes^[^ ^11^ ^]^ or proteins,^[^ ^12^ ^]^ and surface wettability and porosity can be engineered based on the macromolecular order and processing techniques.^[^ ^13^, ^14^, ^15^ ^]^ OMIECs offer a biocompatible interface that facilitates the transduction of electronic signals during oxidation of glucose by GOx. A particularly successful material for enzyme‐based glucose sensing is poly(3,4‐ethylenedioxythiophene) polystyrene sulfonate (PEDOT:PSS), whose ubiquitous fame in the field of bioelectronics is rooted in its commercial availability.^[^ ^16^ ^]^ Organic electrochemical transistors (OECTs) represent an exemplary platform in which OMIECs such as PEDOT:PSS, combined with GOx as the sensing material, show an electronic output varying with exposure to various glucose concentrations.^[^ ^17^, ^18^, ^19^, ^20^, ^21^, ^22^ ^]^ Recently, n‐type (electron‐transporting) OMIECs have been applied as the OECT channel and gate, exhibiting an abrupt increase in the channel current upon GOx/glucose reaction and a very low limit of detection in the absence of a redox shuttle.^[^ ^23^, ^24^ ^]^ However, the use of enzymes in the development of scalable biological sensor systems has encountered criticism due to concerns related to protein instability,^[^ ^25^ ^]^ gradual leaching over time, sensitivity to factors such as temperature, humidity, toxic chemicals, ionic detergents and pH,^[^ ^26^ ^]^ as well as the intricate procedures needed for enzyme immobilization on surfaces.^[^ ^27^ ^]^ In addition, reliance on enzymes hinders the development of adaptable and scalable systems that can be customized for a broader range of molecular analytes that may not have readily available or practically feasible enzymes associated with them.

Consequently, there is growing interest in the exploration of enzyme‐free sensing methods. Glucose sensors have been demonstrated featuring copper oxides, which catalyze the electro‐oxidation of glucose, in conjunction with high surface area electrode materials such as nanoporous gold,^[^ ^28^ ^]^ graphene^[^ ^29^ ^]^ and carbon nanotubes;^[^ ^30^ ^]^ and efficiency has been further improved by applying synergistic co‐catalysts such as cobalt oxides,^[^ ^31^, ^32^, ^33^, ^34^ ^]^ used for faster electron transport pathways.^[^ ^35^ ^]^ Meanwhile, the reversible binding of boronic acid species to 1,2‐ and 1,3‐diols has paved the way for their incorporation in materials as the receptor unit to build enzyme‐free sensors. Due to the synthetic pliability of organic materials, adaptations to the parent structure allow boronic acids to be applied in a variety of glucose‐sensing frameworks. For example, small molecule bis‐boronic acids have been applied as fluorescent sensors^[^ ^36^ ^]^ while electrochemical approaches include the incorporation of the motif into: anthracene‐based immobilized electrodes;^[^ ^37^ ^]^ hydrogels whose permittivity changes upon glucose binding;^[^ ^38^ ^]^ and field‐effect transistors based on copolymer gels^[^ ^39^ ^]^ or surface‐functionalized single‐walled carbon nanotubes.^[^ ^40^ ^]^

Despite their promise as synthetically adaptable semiconductors, OMIECs have been under‐explored for enzyme‐free sensing. A few examples involve composites such as Ni(OH)_2_‐functionalized reduced graphene oxide combined with PEDOT,^[^ ^41^ ^]^ or PEDOT:PSS combined with phenylboronic acid‐functionalized polyacrylamide hydrogels.^[^ ^42^, ^43^ ^]^ These studies demonstrate that enzyme‐free glucose detection using OMIECs is feasible; however, blending different components with OMIECs lead to phase segregation, which necessitates meticulous control of the intercomponent interfaces. Moreover, achieving both sensitivity and selectivity while maintaining device‐to‐device reproducibility remains a challenging task for such composite systems.

**Scheme 1:**
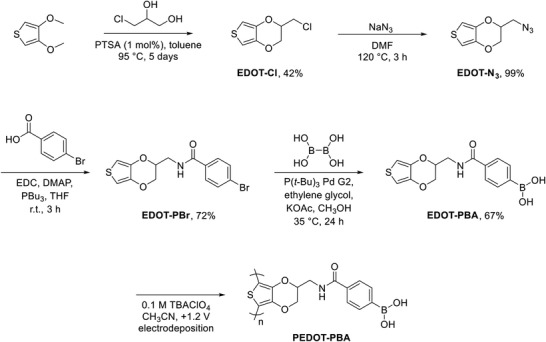
Synthetic route toward the boronic acid‐functionalized polymer PEDOT‐PBA. PTSA = p‐toluenesulfonic acid; DMF = N,N‐dimethylformamide; EDC = 1‐ethyl‐3‐(3‐dimethylaminopropyl)carbodiimide; DMAP = 4‐dimethylaminopyridine; THF = tetrahydrofuran; P(t‐Bu)_3_ Pd G2 = chloro[(tri‐tert‐butylphosphine)−2‐(2‐aminobiphenyl)] palladium(II); TBAClO_4_ = tetrabutylammonium perchlorate.

To address these challenges and leverage the highly tunable structure‐function relationship of OMIECs, we here developed a single active material that combines receptor and transducer functionalities, in this case, the glucose‐binding and electrically conducting moieties, respectively. We have selected 3,4‐ethylenedioxythiophene (EDOT) as an electroactive building block due to its ability to be polymerized electrochemically, thereby avoiding the use of toxic reagents such as the organotin intermediates often used in the conventional synthesis of semiconducting polymers, and enabling the direct functionalization of the OECT gate electrode.^[^ ^44^ ^]^ From an organic design perspective, EDOT is widely considered challenging to modify synthetically; over the last several years, several research groups have contributed pioneering approaches to its structural functionalization, often by exploring alternative core designs such as PheDOT^[^ ^45^ ^]^ and ProDOT,^[^ ^46^, ^47^ ^]^ or refining synthetic strategy to introduce pendant functional groups to the bridging ethylenedioxy moiety.^[^ ^48^, ^49^, ^50^, ^51^, ^52^ ^]^ Of particular note to this work is the demonstration of an EDOT species covalently grafted to a phenylboronic acid moiety using a combination of flexible ether and amide linkages, first presented by Shen et al. in 2018.^[^ ^53^ ^]^ Huang et al. demonstrated sensitivity of this material toward glucose when electrodeposited as the glucose‐binding active layer for electrochemical sensing procedures, such as quartz crystal microbalance (QCM) and electrochemical impedance spectroscopy (EIS), with a limit of detection of 50 µm by the latter technique.^[^ ^54^ ^]^ The monomer was synthesized in three steps from the expensive hydroxymethyl EDOT building block, EDOT‐OH_._^[^ ^53^, ^55^ ^]^ We have previously demonstrated a robust methodology for a shorter and more rigid covalent attachment of analyte‐binding groups to EDOT from the more affordable starting material 3,4‐dimethoxythiophene.^[^ ^56^ ^]^ Here, we harness the same versatile synthetic strategy to develop a scalable phenylboronic acid‐functionalized monomer, **EDOT‐PBA** (**Scheme** **1**). Taking advantage of the strong interaction between **EDOT‐PBA** and hexose sugars, we explore two approaches to create a functional **PEDOT‐PBA** film as the gate electrode material for the OECT‐based sensor. First, we fabricate a **PEDOT‐PBA** film through a standard electropolymerization, which exhibits high sensitivity to glucose in the range of 10 µm to 10 mm. Second, our rigid monomer design allows us to introduce a molecular imprinting technique to electropolymerize an *imprinted* **PEDOT‐PBA** film, resulting in a more reliable sensing signal characterized by a smaller standard deviation, lower limit of detection and faster response upon exposure to glucose.

## 2 Results and Discussion

### 2.1 Synthesis and Characterization

The design approach toward a novel conjugated polymer featuring a pendant boronic acid was adapted from our previous work^[^ ^56^ ^]^ modifying EDOT using the Staudinger‐Vilarrasa^[^ ^57^, ^58^ ^]^ reaction. Demonstrating the versatility of the same building block, **EDOT‐N_3_**, herein we introduce 4‐bromobenzoic acid to afford the bromide intermediate **EDOT‐PBr** in good yield, followed by an adapted Miyaura borylation^[^ ^59^ ^]^ to give the free boronic acid **EDOT‐PBA** without requiring a protecting group (Scheme 1). Detailed synthetic procedures are provided in the Supporting Information.

**Figure 1:**
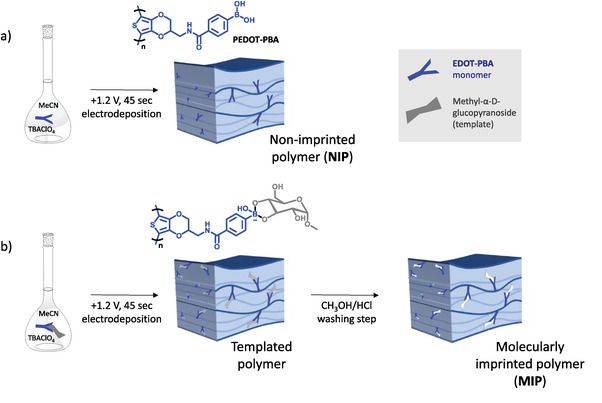
Illustration of the preparation of PEDOT‐PBA as a) a non‐imprinted polymer (NIP) film and b) the molecularly imprinted polymer (MIP) film, templated with methyl α‐D‐glucopyranoside and subsequently washed with 1:1 CH_3_OH:0.05 m aq. HCl (30 min) and H_2_O (10 min) for template removal.

The corresponding polymer, **PEDOT‐PBA**, was synthesized using potentiostatic electrodeposition, allowing us to obtain a thin film of **PEDOT‐PBA** at the working electrode. Rather than exploring different deposition conditions (choice of solvent, deposition method and parameters), our approach to judicious nanostructuring of the polymer film is instead via the development of a molecularly imprinted polymer (MIP) using electropolymerization in the presence of a sugar template (**Figure** **1**). Since boronic acids are well established to bind a range of 1,2‐ and 1,3‐diols,^[^ ^60^ ^]^ we investigate whether the MIP framework may be used to improve selectivity toward a specific sugar, glucose. The framework of a MIP consists of a porous, 3D polymer matrix that contains size‐ and shape‐specific cavities for the analyte as a result of polymerization with the template analyte in situ. While classical MIPs are aliphatic and can be featured as receptors in multi‐component sensor devices, such as those for glucose,^[^ ^61^, ^62^, ^63^ ^]^ it is possible to create these systems in conjunction with a semiconducting material as the backbone,^[^ ^64^, ^65^, ^66^ ^]^ combining receptor and transducer into one component to enable incorporation as the active layer in an electrochemical device. An important consideration for MIP fabrication is the rigidity of the resulting framework, which ensures the retention of cavity shape and size upon template removal. In classical (non‐conjugated) MIPs, this is usually achieved by the addition of a cross‐linker.^[^ ^67^ ^]^ However, many examples in the literature of selective recognition via templated polymerization of conjugated materials, self‐described as “molecularly‐imprinted”, omit the cross‐linker from their composition,^[^ ^64^, ^66^, ^68^, ^69^, ^70^, ^71^ ^]^ as rigidity can be conferred from the backbone of the conjugated material.^[^ ^65^ ^]^ With this in mind, our synthetic design features a short, conformationally restrictive phenyl/amide bridging linkage between glucose‐binding boronic acid and electropolymerizable EDOT moieties.

While MIPs templated from glucose have been incorporated into a variety of composite devices to act as selective sensors for the same material,^[^ ^63^, ^72^, ^73^, ^74^, ^75^ ^]^ here, we employ a methylated analogue of glucose, methyl α‐D‐glucopyranoside, which has precedent in this application for glucose sensing,^[^ ^76^ ^]^ and has additional benefits such as solubility in acetonitrile for deposition, and reducing uncertainty in the measurement of the analyte concentration as a result of template leakage.^[^ ^77^, ^78^, ^79^ ^]^

While glucose‐boronic acid binding is well‐established in an aqueous environment at a basic pH,^[^ ^80^, ^81^ ^]^ it was important in this case to confirm that **EDOT‐PBA**:template binding occurs within the organic medium used for polymer deposition, specifically, acetonitrile, before it could be used as the templated system. Note that methyl α‐D‐glucopyranoside is not soluble in acetonitrile up to 5 mM in isolation or alongside unmodified EDOT. However, it is fully soluble at this concentration in the presence of the **EDOT‐PBA** binding monomer, allowing the electrodeposition of uniform polymer films to occur (Figure S1, Supporting Information). Shielding of the polar hydroxyl groups on the sugar may, therefore, be occurring as a result of the binding interactions with the boronic acid moiety of **EDOT‐PBA**. To investigate this, equimolar or 2:1 molar solutions of **EDOT‐PBA** and methyl α‐D‐glucopyranoside were prepared in deuterated acetonitrile and examined by ^1^H NMR spectroscopy. A detailed analysis of the results, given in the Supporting Information, demonstrates that a clear binding interaction is occurring between **EDOT‐PBA** and methyl α‐D‐glucopyranoside in acetonitrile, evidenced by features such as the quenching of free boronic acid protons at 6.15 ppm in the presence of the template (Figure S3, Supporting Information) as well as the change in chemical shift to several proton environments on the sugar in the presence of the monomer (Figures S2 and S4, Supporting Information). Another important step in MIP fabrication is the removal of the template from the matrix to create analyte‐specific cavities. Since the binding of the boronic acids with sugars is pH‐dependent, methyl α‐D‐glucopyranoside can be removed under acidic conditions. Based on previous work, this was achieved by submersion in 1:1 MeOH:50 mm HCl (aq.) for 30 min with gentle stirring followed by 10 min in deionized water.^[^ ^82^ ^]^

With all of the aforementioned parameters in mind, the procedure for electrodeposition can be summarized as follows: for the non‐imprinted polymer (NIP), a voltage of +1.2 V was applied to a 10 mm solution of the monomer in acetonitrile, alongside 0.1 m tetrabutylammonium perchlorate electrolyte, in a three‐electrode setup with Ag/AgNO_3_ reference and Pt counter electrodes. For the MIP, the procedure is identical with the additional presence of 5 mm methyl α‐D‐glucopyranoside, and is followed by the washing step detailed above (Figure 1). The type of working electrode, which becomes the substrate for the polymer film, can be selected based on intended application. Here, we use indium tin oxide (ITO)‐glass, a Ti‐Au‐coated piezoelectric sensor, or Cr‐Au on either polyimide, glass or Si where appropriate for UV–vis, QCM‐D, electrochemical measurements, OECT, and scanning electron microscopy respectively.

### 2.2 High‐Resolution Scanning Electron Microscopy

**Figure 2:**
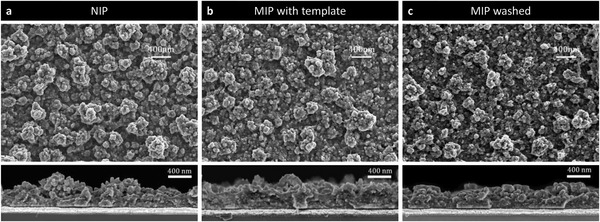
Top view (top) and cross section (bottom) HRSEM images of PEDOT‐PBA polymerized by potentiostatic electrodeposition (+1.2 V, 15 s, from 10 mm monomer with 0.1 m TBAClO_4_ in acetonitrile) on highly doped++ 525 µm Si coated with 10 nm/100 nm CrAu. a) NIP only; b) MIP templated with 5 mm methyl α‐D‐glucopyranoside, before washing; c) MIP templated with 5 mm methyl α‐D‐glucopyranoside then washed with 1:1 MeOH:50 mm HCl (aq.) for 30 min. All scale bars are 400 nm.

To confirm the morphological differences between the differing polymer architectures, high‐resolution scanning electron microscopy (HRSEM) was conducted on thin films of the following: NIP, templated MIP (unwashed; template present), and washed MIP (template removed). The top‐view images in **Figure** **2** reveal that all films exhibit particle‐aggregate pillars on the film surface, with a less dense morphology observed for the imprinted polymer. Examining the cross section, we note that the templated and washed MIP films (Figure 2b,c) display superior uniformity compared to the NIP films (Figure 2a). We note that the washing step of the MIP results in reduced surface roughness, which corresponds to a reduction in particle‐aggregate size; the resultant smoothing and thinning of the film upon washing can be visualized clearly in the cross section images. It also appears that the MIP film somewhat contracts during the washing step. We, therefore, speculate that further improvements in the **PEDOT‐PBA** MIP sensing platform could be achieved through a more rigorous control of the MIP architecture and its structural robustness. Moreover, to investigate the structural features of these film architectures, we carried out the spectroelectrochemical analysis of thin films of **PEDOT‐PBA** deposited onto ITO‐coated glass. The results, detailed in Figures S5 and S6 (Supporting Information), indicate more disordered polymer backbone conformations for the MIP film, compared to the corresponding NIP film, likely a result of the non‐covalent template incorporation during the electrodeposition.

### 2.3 Electrochemical Properties

**Figure 3:**
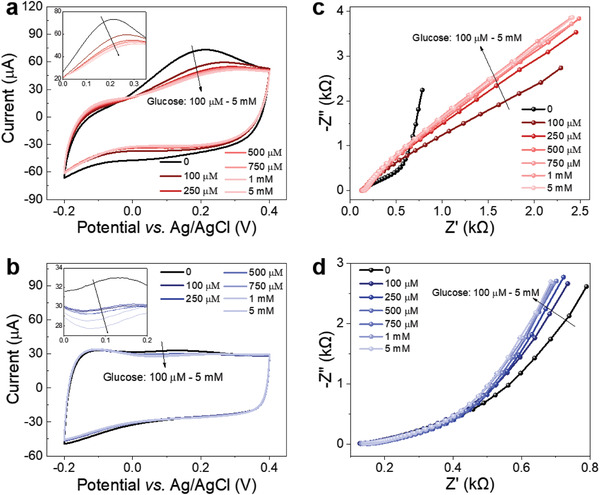
Electrochemical measurements of PEDOT‐PBA (NIP) and imprinted PEDOT‐PBA (MIP) polymer films in PBS electrolyte. The CV curves of the a) NIP and b) MIP before and after interactions with glucose at different concentrations. The scan rate was 50 mV s^−1^ and the voltage window for forward scan was from −0.2 to 0.4 V versus Ag/AgCl reference electrode. Nyquist plots of c) NIP and d) MIP polymer films before and after interactions with glucose at different concentrations. The EIS measurements were conducted at 0 V versus the open‐circuit potential.

We first characterized the electrochemical behavior of the **PEDOT‐PBA** films polymerized on Au substrates using cyclic voltammetry (CV) and EIS. The cyclic voltammogram of the **PEDOT‐PBA** (NIP) electrode in phosphate‐buffered saline (PBS) reveals characteristics of pseudocapacitive behavior with oxidation (forward scan) and reduction (reverse scan) peaks, whereas the CV curve of the imprinted **PEDOT‐PBA** (MIP) exhibits a more rectangular shape, indicating a more capacitive behavior. Next, CV curves were recorded for the NIP and MIP‐coated electrodes, which had been incubated in PBS containing glucose for 1 min, with this experiment subsequently repeated with increasing glucose concentrations from 100 µm to 5 mm. As glucose concentration increased (**Figure** **3**a,b), the area under the CV curves for both polymer electrodes decreased. This observation suggests that the binding of glucose to the PBA units within the polymer films reduces their capacitance. Notably, our previous study demonstrated that a similar PEDOT film that lacks the PBA unit did not respond to the addition of glucose in PBS, emphasizing the essential role of PBA units in glucose interaction and its subsequent impact on the electrochemical properties of the polymers.^[^ ^42^ ^]^

A similar conclusion can be reached by analyzing the electrochemical impedance spectra of the films. The Nyquist plots in Figure 3c,d show changes that scale with the increase in glucose concentrations, particularly at the low frequency (0.1 to 1 Hz) regime, for both films. We used electrical circuit models, shown in Figure S7 (Supporting Information), to analyze these spectra and found that both films show a capacitance decrease upon glucose incubation (Table S1, Supporting Information). Note, however, that a quantitative comparison between the two films could not be made as two different models had to be used for a reasonable fit quality. The reduction in capacitance, as deduced from CV and EIS analysis, can be attributed to the PEDOT structure becoming more compact upon binding of glucose molecules to PBA units, restricting the movement of ions within the film.

### 2.4 Transistor Characterization

**Figure 4:**
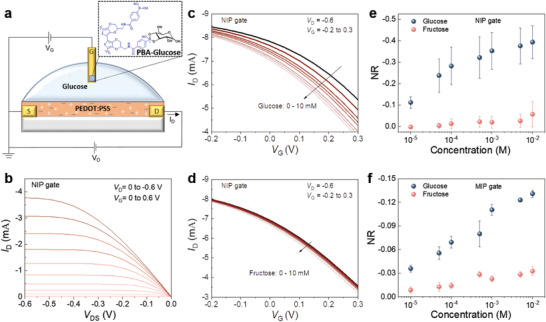
The OECT biosensor configuration and characteristics. a) The **PEDOT‐PBA** functionalized Au electrode is used as the gate electrode of the OECT with a PEDOT:PSS film in the channel. The channel has a width of 10 µm and length of 100 µm and the gate electrode is 2 mm × 2 mm. The complexation of the PBA unit with glucose molecules occurs at the gate electrode, decreasing the capacitance of the gate electrode, thereby modulating the OECT conductance. The measurement and analyte solution is 1X PBS. b) The output characteristics of the OECT with the NIP electrode as the gate. c,d) The transfer characteristics of the OECT channel gated with the NIP electrode exposed to glucose and fructose solutions of varying concentrations, respectively. The NR response of OECT biosensor gated with e) the NIP electrode or f) the MIP electrode. Error bars represent the standard deviation from measurements conducted using 3 gate electrodes. The NR values were extracted at *V*_D_ = −0.6 and *V*_G_ = 0.3 V.

While the films show small changes to their electrochemical properties by CV and EIS upon glucose‐PBA complexation, the molecular design of **PEDOT‐PBA** leads to a further advantage. The ability to electropolymerize (with or without molecular imprinting) while retaining semiconducting behavior in an aqueous environment enables the application of the NIP and MIP directly as gate materials in the OECT configuration. OECTs, owing to their high transconductance (*g*_m_) feature, are effective at the transduction of weak voltage fluctuations, associated with biological events occurring in an aqueous medium in the vicinity of the gate electrode, into large current signals.^[^ ^23^, ^83^ ^]^ The OECT can therefore be used to monitor polymer‐target interactions in a simulated biological environment. **Figure** **4**a illustrates the investigated OECT device structure, featuring a PEDOT:PSS channel gated by the **PEDOT‐PBA** electrode. In this device configuration, we are using the OECT as a transconductance amplifier: the formation of the PBA‐glucose complex at the gate electrode is envisaged to alter the capacitance of the gate electrode, which will be translated into changes in the channel current. The separation of the gate electrode from the channel allows us to reuse the channel with various gate electrodes that can be disposed. In this configuration, only the gate electrode is exposed to the analyte solution, while the channel can be operated many times. The gate electrode could also be fabricated next to the channel, in this case, we expect the sensor performance to be even higher by use of microfluidics.^[^ ^84^ ^]^

We first assessed the performance of the NIP and MIP as the gate electrode and found that both can effectively modulate the PEDOT:PSS channel (Figure 4b; Figure S8, Supporting Information). Figure 4c,d presents the steady‐state performance of the OECT with NIP‐gated electrodes after exposure to glucose or fructose solutions at varying concentrations. The transfer curves of the OECT reveal a consistent reduction in the source‐drain current (*I*_D_) as the glucose concentration increases (Figure 4c). In contrast, the changes are minimal when the gate is exposed to fructose (Figure 4d). Figure S8 (Supporting Information) shows the same data extracted from the experiments performed with the MIP‐gated OECT. Glucose binding lowers the capacitance of **PEDOT‐PBA** gate electrodes, hence their capability to dope and dedope the channel. Therefore, we observe a smaller *I*_DS_ at all gate voltages at different polarities upon glucose binding. Figure 4e,f depicts the calibration curve for the OECT biosensors, with the normalized response (NR) value calculated from the variations in *I*_D_ in response to analyte concentrations that range from 100 µm to 10 mm. Both NIP and MIP gate electrodes exhibit a much higher response to glucose than to fructose, indicative of the preferential binding of PBA to glucose. The statistical data of the high R^2^ value and the linear equation of the calibration plots are shown in Table S2 (Supporting Information), indicating a high degree of linearity and thus a good correlation between the sensor output and glucose concentrations within the tested range. Limit of detection (LOD) values were extracted from this analysis using the formula LOD = 3.3 × (σ/S), where σ represents the standard deviation of the y‐intercept, and S is the slope of the calibration curve. The resulting LOD values are 28.2 and 22.3 µm for the NIP and MIP respectively, which are among the lowest of recent non‐enzymatic, PBA‐based electrochemical glucose sensors (Table S3, Supporting Information). We conducted a specificity test and recorded the device current change in response to other common interferents or abundant molecules in serum, such as lactate, cholesterol, and human serum albumin (Figure S9a, Supporting Information). These species generated a change in MIP‐based‐OECT current. However, the magnitude of the interfering signal was much lower than compared to the sensor's response to glucose. In addition, the MIP sensors also successfully detected glucose contained in the commercial serum sample as well as the additional spiked amount of glucose, indicating the sensor's capability in complex biological conditions (Figure S9b, Supporting Information). We also conducted a series of experiments that evaluated the sensor performance when the gate was exposed to target solutions at different ionic strengths, pH levels, or temperatures. The results are summarized in Figure S10a–c (Supporting Information) and indicate that the MIP film maintains its sensitivity to glucose under different environmental conditions. While the NR values changed, the sensitivity remained similar at higher temperatures or lower ionic strength conditions. A lower pH (6) somewhat affected the dynamic range, and overall, under these conditions, the sensor maintained its sensitivity, suggesting the stability of the film.

Although the NIP gate electrode shows a higher NR value compared to the MIP gate electrode, further analysis of the data reveals advantages offered by the MIP electrodes: i) A reduced limit of detection. ii) A significantly smaller standard deviation, indicating better reproducibility among different electrode batches. iii) A single linear response within a broad range of glucose concentrations (10 µm to 10 mm), with a sensitivity slope of −0.03, as opposed to the NIP electrode's two linear regions (with a slope of −0.17 for 10 to 100 µm and −0.05 for 100 µm to 10 mm), which suggests a more straightforward analysis in a broader detection range. Despite the lower NR, the smaller standard deviation and linear change in current especially at high glucose concentrations make MIP superior to NIP. For MIP sensors, it is possible to assign each current reading to one glucose level, whereas for NIP above 10^−4^ m, it is challenging to estimate glucose concentrations. These results underscore the capability of a single‐component **PEDOT‐PBA** electrode, whether in the form of our NIP or MIP architecture, to selectively capture the glucose molecules and function in OECT biosensing platforms. The integration of the **PEDOT‐PBA** electrode into the OECT offers an amplified response compared to a three‐electrode system, and it holds the potential for further development into miniaturized and integrated sensors.

### 2.5 QCM‐D Analysis of the Speed and Amount of Glucose Uptake

**Figure 5:**
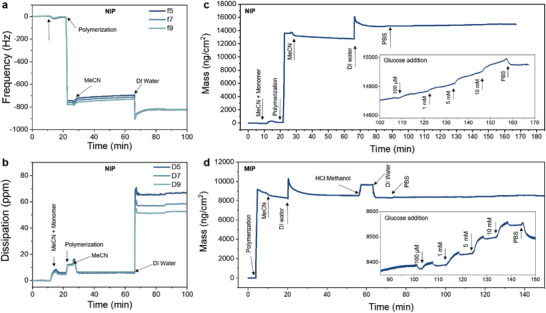
QCM‐D monitoring of the electrodeposition of PEDOT‐PBA films and their glucose sensing performance. The change in a) the frequency (f) and b) the dissipation (D) signals plotted during the polymerization of the NIP film using the 5^th^, 7^th^, and 9^th^ overtones. c,d) show the calculated mass changes on the sensors during the electrodeposition of NIP and MIP films. The inset figures in (c) and (d) show the corresponding mass uptake after the electrodeposition step as the glucose concentration is increased in the solution.

We used the quartz crystal microbalance with dissipation monitoring (QCM‐D) technique to dynamically observe the real‐time formation of both NIP and MIP films. QCM‐D is a non‐invasive gravimetric approach for tracking adsorption and desorption processes on film‐coated quartz crystal sensors. **Figure** **5**a,b presents the raw QCM‐D profiles, illustrating the dynamic changes in frequency and dissipation signals before and after the in situ electrochemical polymerization of the NIP film on the quartz crystal sensor. Using the Sauerbrey equation, we translated the change in frequency into deposited mass, revealing that 12 618 ng cm^−2^ of NIP film was generated during electropolymerization under our specific fabrication conditions (1.2 V for 45 s) (Figure 5c). The simultaneous increase in dissipation signal (Figure 5b) indicates a softening of the QCM‐D sensor, highlighting the inherent soft nature of deposited polymer films. The polymerization of MIP resulted in a lower deposited mass of 8294 ng cm^−2^, which subsequently experienced a mass loss of ≈306 ng cm^−2^ following the washing step. This decrease in mass upon washing indicated the successful removal of the glucose template (methyl α‐D‐glucopyranoside) from the polymer matrix. It is worth noting that the lighter MIP deposited under the same conditions as the NIP could be attributed to the presence of the nonconducting glucose template during the polymerization process. This presence likely resulted in lower polymer yields and, hence, fewer available PBA units within the polymer matrix. The reduction in the number of active binding sites may explain the lower NR observed for **PEDOT‐PBA** MIP compared to the NIP.

The QCM‐D analysis was used to monitor the interaction of glucose with NIP and MIP films (insets of Figure 5c,d). When glucose was introduced into the polymer films, a decrease in frequency was detected. The magnitude of this frequency reduction increased as we incubated the films with increasing glucose concentrations. The increase in film mass is primarily attributed to glucose uptake, facilitating the formation of the glucose‐PBA complex within the polymer matrix. Our calculations indicated that an additional mass of 79 and 48 ng cm^−2^ were adsorbed when a 10 mm glucose solution was introduced to the NIP and MIP films, respectively, assuming that the entire adsorbed mass was linked to the glucose‐PBA complex. Despite the lower amount of glucose uptake in the MIP film, which we relate to fewer accessible binding sites, this film achieved faster signal stabilization during incubation with glucose. The faster and more stable signal may also be associated with the smaller standard deviation observed among the MIP sensors. In summary, the QCM‐D results revealed that the **EDOT‐PBA** monomers were successfully polymerized in the form of NIP and MIP films, with both films interacting with glucose molecules. The QCM‐D also suggests that the fabrication method of the MIP film could be further optimized, such as by using different cross‐linker structures as well as a variety of electrochemical deposition conditions to achieve a thicker and more rigid deposited polymer film with a higher number of PBA units, potentially enhancing the binding capacity of the MIP sensor.

## 3 Conclusion

In summary, we present a non‐enzymatic glucose detection platform using a newly synthesized functionalized monomer, **EDOT‐PBA**. This monomer features a simple yet effective synthetic design by combining electrically conducting moieties and receptor units in a single component without the need for further chemical conjugation. The electrodeposition technique was successfully demonstrated to produce functionalized polymer films using two scenarios, i) pristine **PEDOT‐PBA** (NIP), and ii) imprinted **PEDOT‐PBA** (MIP). Both functionalized polymer architectures are shown to effectively enable glucose binding and signal transduction, as demonstrated through various methods including CV, EIS, OECT, and QCM‐D. The incorporation of the molecularly imprinted polymer (MIP) architecture introduced several advantageous features compared to the non‐imprinted counterpart, such as enhanced film uniformity, as observed by high‐resolution SEM, which significantly improves the reproducibility in the NR response, as indicated by a very low standard deviation of OECT output signal. Additionally, the MIP film results in a lower limit of detection, faster stabilization upon glucose uptake, and broader linear response, as indicated by QCM‐D and single sensitivity response in the range of 10 µm to 10 mm. We anticipate that further improvements of the MIP sensing platform can be achieved by carefully increasing the structural robustness of the polymer film without significantly compromising the flexibility required to create template‐specific cavities. This could be achieved for instance using structurally similar cross‐linkers to prevent the observed change in microstructure upon template removal. We furthermore notice the modularity of the system; tailoring toward glucose sensing was achieved through the choice of template, meaning that other polar biomarkers could be targeted with the same system solely by changing the template molecule. In conclusion, this versatile and adaptable synthetic design framework holds great promise for further optimization and broader application to a wide range of other analytes.

## 4 Experimental Section

### Materials

Unless otherwise stated, all chemical synthesis was carried out using commercially available reagents, used as supplied from Sigma Aldrich, Acros Organics, Alfa Aesar, or Fluorochem, in combination with solvents from Honeywell. Anhydrous solvents were purchased from Acros Organics or collected from an MBRAUN MB SPS‐800 solvent purification system. 3,4‐Ethylenedioxythiophene (EDOT), (3‐glycidyloxypropyl)trimethoxysilane (GOPS), sodium dodecylbenzenesulfonate (DBSA), ethylene glycol (EG), d‐(+)‐glucose, d‐(+)‐fructose, and 1X phosphate‐buffered saline (PBS) were obtained from Sigma‐Aldrich and utilized without any further modification. 1X PBS (pH 7.4) solution contains 137.9 mm sodium chloride (NaCl), 2.7 mm potassium chloride (KCl), 1.5 mm potassium phosphate monobasic (KH_2_PO_4_), and 8.1 mm sodium phosphate dibasic (Na_2_HPO_4_) anhydrous. All aqueous solutions were prepared using ultrapure water from Millipore Milli‐Q. Solutions of glucose and fructose with varying concentrations were prepared in PBS. The channel of OECTs was fabricated from a dispersion of PEDOT:PSS (PH1000, Heraeus) incorporating GOPS, DBSA, and EG.

### Synthesis

Where it was indicated that anhydrous conditions were used, reactions were carried out in a sealed environment under a nitrogen atmosphere, using glassware oven‐dried at 120 °C overnight. Analytical thin layer chromatography was carried out on Merck Kieselgel 60 aluminum‐backed silica plates, with visualization using short‐wave ultraviolet light, or a potassium permanganate or phosphomolybdic acid stain where appropriate. Column chromatography was carried out using VWR silica gel (40–60 µm). Full synthetic procedures and characterization for **EDOT‐PBA** are detailed in the Supporting Information.

### Fabrication of the PEDOT‐PBA Films

The synthesis of **PEDOT‐PBA** was conducted using electrochemical polymerization in an organic solvent (acetonitrile) at a constant voltage (potentiostatic mode). Two different pre‐monomer dispersions were prepared for this study, i) pristine **EDOT‐PBA** (referred to as Non‐Imprinted Polymer, NIP), and ii) **EDOT‐PBA** mixed with glucose template (referred to as Molecularly Imprinted Polymer, MIP). A detailed composition of each dispersion is shown in **Table** **1**. The monomer dispersion was placed into the electrochemical cell in a three‐electrode configuration using a Pt counter electrode, an Ag/AgNO_3_ reference electrode for organic solvent, and Au working electrode. The choice of substrate for the working electrode varies depending on the specific measurements being conducted, for instance, Cr‐Au‐coated Kapton was used for electrochemical measurement and the gate electrode of organic electrochemical transistors (OECTs), while ITO‐coated glass was used for UV–vis spectroscopy, and a Ti‐Au‐coated piezoelectric sensor was used for Quartz Crystal Microbalance with Dissipation Monitoring (QCM‐D). The electrochemical polymerization was performed at a voltage of +1.2 V for a duration of 45 s. Following the process of electropolymerization, the coated electrodes were rinsed using deionized (DI) water in order to remove any excess unreacted molecules and then dried with N_2_ gas spray.

**Table 1:** Composition of monomer dispersions.

Chemicals	NIP	MIP
EDOT:PBA	10 mm	10 mm
TBAClO_4_	100 mm	100 mm
Methyl α‐D‐glucopyranoside	–	5 mm
Acetonitrile	5 mL	5 mL

### Electrochemical and Spectroelectrochemical Measurements

The electrochemical properties of the **PEDOT‐PBA** electrodes were evaluated using cyclic voltammetry (CV) and electrochemical impedance spectroscopy (EIS) techniques. These analyses were conducted in a standard electrochemical cell equipped with Ag/AgCl reference electrode, a Pt counter electrode, and a potentiostat (Autolab PGstat128N, MetroOhm). The electrochemical measurements were conducted within a cell that was placed inside a grounded Faraday cage. The electrolyte used in the experiment was PBS, and the samples with different concentrations of glucose or fructose (100 µm to 5 mm) were also prepared in PBS for sample incubation. The CV curves were acquired by sweeping the potential between −0.2 and 0.4 V versus Ag/AgCl with a scan rate of 50 mV s^−1^. The impedance spectra were recorded at a DC voltage of 0 V versus open circuit potential and an AC modulation of 10 mV over a frequency range of 0.1–100,000 Hz. Each measurement was performed in PBS electrolyte following consecutive incubations with increasing concentrations of glucose solutions.

Spectroelectrochemical measurements were carried out in 1X PBS using a PalmSens EmStat3+ potentiostat alongside a Shimadzu UV‐3600 Plus UV–Vis–NIR Spectrophotometer. Working electrodes were ITO‐coated glass purchased from VisionTek Systems (20 mm × 20 mm), cleaned with detergent followed by sonication in H_2_O (3 × 15 min), acetone (2 × 10 min), and isopropyl alcohol (2 × 5 min), followed by plasma cleaning using Harrick PDC‐32G‐2 for 5 min directly before use. Counter electrodes were Pt wire, reference electrodes were Ag/AgCl in H_2_O for aqueous systems, all from BASi. Samples for HRSEM were deposited on 20 mm × 10 mm highly doped++ 525 µm Si coated with 10 nm/100 nm Cr/Au.

### OECT Fabrication and Glucose Sensing Measurements

The OECTs were fabricated using standard photolithography as described in the previous work.^[^ ^42^ ^]^ The width (*W*) of the OECT channel is 100 µm, while its length (*L*) is 10 µm. PEDOT:PSS (PH 1000) was mixed with EG (5 vol%), DBSA (0.002 vol%), and GOPS (1 wt%) and sonicated for 30 min. The dispersion was then filtered through 0.45 µm glass fiber filters and was subsequently spin‐coated on the channel at 1500 rpm for 30 s. Following the spin‐coating process, the Parylene‐C, which served as a sacrificial layer, was peeled off, and the films were annealed at 140 °C for 1 h under ambient conditions. The Au gate electrode was coated with electropolymerized **PEDOT‐PBA** identically, as described above. All measurements were performed in the top gate electrode configuration and 1X PBS as the electrolyte. The steady‐state characteristics of the OECT (output and transfer curves) were recorded using a Keithley 2612A with customized LabVIEW software. For sensing measurements, the NIP and MIP‐coated electrodes were incubated in PBS solution containing glucose for 1 min followed by rinsing in PBS and measured the corresponding IV characteristics. This experiment subsequently repeated with increasing glucose concentrations. The normalized response of OECT channel current was calculated using Equation (1), where *I*_t_ is the current after incubation with the glucose, while *I*_0_ is the output current of the device before exposure to glucose.

(1) $$NR\;=\;\frac{I_{t}-I_{0}}{I_{0}}$$

### Quartz Crystal Microbalance with Dissipation Monitoring (QCM‐D)

QCM‐D measurements were performed using a Q‐sense analyzer (model QE401, manufactured by Biolin Scientific). **PEDOT‐PBA** films were electropolymerized in situ on QCM‐D crystals using a potentiostat (PalmSens) coupled with Q‐sense electrochemistry module. The three‐electrode configuration consisted of Ag/AgCl reference electrode, a Pt counter electrode, and the QCM‐D sensor serving as the working electrode. The measurements were carried out at 24 °C utilizing a peristaltic pump to control the flow rate at 20 µL min^−1^ for electropolymerization process and at 100 µL min^−1^ for glucose sensing. Following the stabilization of QCM‐D signals in PBS, aliquots of glucose solutions were introduced into the chamber and the subsequent glucose absorption was observed by monitoring changes in frequency (Δ*f*). The Sauerbrey equation (Equation (2)) was used to quantify the change in mass (Δ*m*) on the sensor resulting from glucose uptake,

(2) $$\Delta m\;=\;\frac{-17.7}{n}\Delta f_{n}$$

where *n* represents the chosen overtone for the calculations (in this case, *n* = 7), while the constant value of −17.7 is derived from the resonant frequency, active area, density, and shear modulus of the piezoelectrically active quartz crystal.

## Conflict of Interest

The authors declare no conflict of interest.

## Supporting information

Supporting Information

## Data Availability

The data that support the findings of this study are available from the corresponding author upon reasonable request.
