# Supplementary material for: Single‐Component Electroactive Polymer Architectures for Non‐Enzymatic Glucose Sensing
Source: Adv Sci (Weinh). 2024 Mar 23;11(27):2308281. doi: 10.1002/advs.202308281 (PMC11251565; doi:10.1002/advs.202308281)
Supplement: Supplementary file 1 — Supporting Information [file ADVS-11-2308281-s001.pdf]

ADVANCED SCIENCE
Open Access

Supporting Information

for *Adv. Sci.*, DOI 10.1002/advs.202308281

Single-Component Electroactive Polymer Architectures for Non-Enzymatic Glucose Sensing

Christina J. Kousseff, Shofarul Wustoni, Raphaela K. S. Silva, Ariel Lifer, Achilleas Savva, Gitti L. Frey, Sahika Inal and Christian B. Nielsen**

Single-component electroactive polymer architectures for non-enzymatic glucose sensing

Supplementary Information

Christina J. Kousseff,[a] Shofarul Wustoni,[b] Raphaela K. S. Silva,[b] Ariel Lifer,[c] Achilleas Savva,[d] Gitti L. Frey,[c] Sahika Inal,[b*] Christian B. Nielsen[a*]

[a]*Department of Chemistry, Queen Mary University of London, Mile End Road, London E1 4NS, UK*

[b]*Organic Bioelectronics Laboratory, Biological and Environmental Science and Engineering, King Abdullah University of Science and Technology (KAUST), Thuwal 23955-6900, Saudi Arabia*

[c]*Department of Materials Science and Engineering, Technion–Israel Institute of Technology, Haifa 32000, Israel*

[d]*Bioelectronics Section, Dept. of Microelectronics, Faculty of Electrical Engineering, Mathematics and Computer Science (EEMCS), Delft University of Technology, 2628 CD Delft, The Netherlands*

General experimental

^{1}H NMR spectra were recorded at 400 MHz on a Bruker Avance III spectrometer. Chemical shifts (δ) are quoted to the nearest 0.01 ppm relative to tetramethylsilane, with the residual solvent peak used as the internal standard: $CHCl_3$ (7.26 ppm), DMSO (2.50 ppm) or CH_3CN (2.10 ppm). Coupling constants (*J*) are given to the nearest 0.1 Hz. Peak multiplicities for resonances are noted as: s, singlet; d, doublet; dd, doublet of doublets; t, triplet; q, quartet; m, unresolved multiplet. ^{13}C NMR spectra were recorded at 101 MHz on a Bruker Avance III spectrometer. Chemical shifts (δ) are quoted to the nearest 0.1 ppm, with reference to the given solvent $CDCl_3$ (77.0 ppm) or DMSO-d_6 (39.5 ppm) as the internal standard.

All mass spectra were obtained from the analytical laboratory in the Department of Chemistry at Queen Mary University of London as follows: low resolution mass spectra were obtained to 1 d.p. from either an Agilent 1100 Series Liquid Chromatograph with a SL Ion Trap mass selective detector, or an Agilent 6890N Gas Chromatograph with an Agilent 5973N mass selective detector, or a Bruker Autoflex MALDI-TOF spectrometer. High resolution mass spectra were obtained to 4 d.p. from a Waters Synapt G2-Si High-Definition Mass Spectrometer. Melting points were obtained using a Stuart SMP11 melting point apparatus and are uncorrected.

Synthesis

Note: compounds **EDOT-Cl** and **EDOT-N_3** are the same intermediates reported in our previous work;[1] for completeness, syntheses and characterization data are reproduced here.

2-(Chloromethyl)-2,3-dihydrothieno[3,4-*b*][1,4]dioxine **EDOT-Cl**

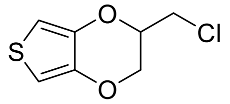

Under an atmosphere of nitrogen, 3,4-dimethoxythiophene (5.0 g, 35 mmol, 1.0 eq.), 3-chloro-1,2-propanediol (7.25 mL, 88 mmol, 2.5 eq.) and *p*-toluenesulfonic acid monohydrate (66 mg, 0.35 mmol, 1 mol%) were dissolved in anhydrous toluene (140 mL) in a 250 mL 2-necked round bottom flask equipped with distillation apparatus. The solution was heated at 95 °C for 24 h, over which time a black oily precipitate was formed. After this time, another equal portion of the diol (7.25 mL, 88 mmol, 2.5 eq.) was added and the mixture was stirred at the same heat for a further 4 days, then cooled to room temperature. The toluene solution was decanted from an insoluble dark blue oil and concentrated *in vacuo*. The crude material was purified by chromatography over silica gel, with the product **EDOT-Cl** eluting in 7:3 hexane:DCM as a white solid (2.76 g, 42%). $R_f = 0.25$

^{1}H NMR (400 MHz, $CDCl_3$): δ 6.36 (s, 2H), 4.40-4.34 (m, 1H), 4.28 (dd, 1H, *J* = 11.7 Hz, *J* = 2.2 Hz), 4.15 (dd, 1H, *J* = 11.7 Hz, *J* = 6.2 Hz), 3.75-3.64 (m, 2H). **^{13}C NMR** (101 MHz, $CDCl_3$): δ 141.3, 140.8, 100.3 (× 2), 73.0, 65.7, 41.5. **GCMS** (EI): *m/z* calcd. for $C_7H_7O_2ClS$ (M^+) 190.0; found 190.0. **mp:** 41-43 °C, in accordance with literature.[2,3]

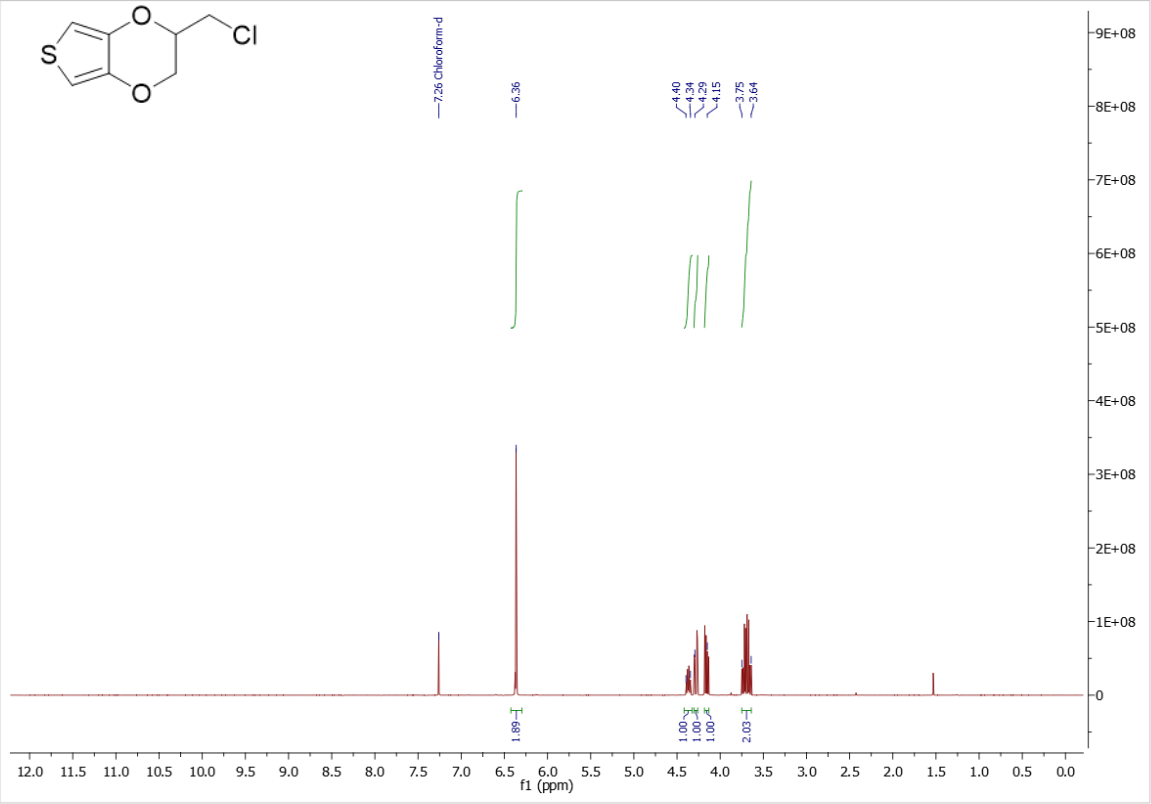
S
O
O
Cl
7.26 Chloroform-d
6.36
4.40
4.34
4.29
4.15
3.75
3.64
1.89
1.00
1.00
1.00
2.03
f1 (ppm)

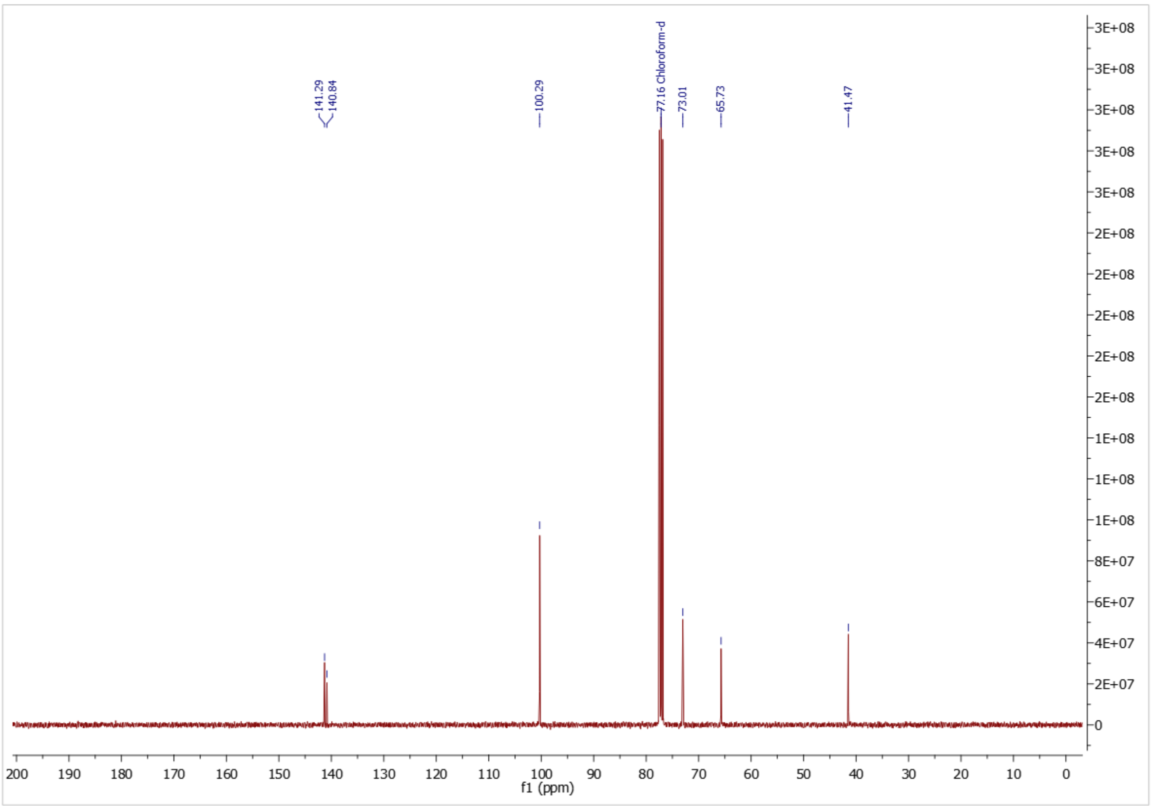
141.29
140.84
100.29
77.16 Chloroform-d
73.01
65.73
41.47
f1 (ppm)

2-(Azidomethyl)-2,3-dihydrothieno[3,4-*b*][1,4]dioxine **EDOT-N_3**

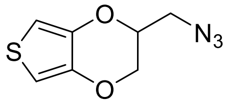

Under an inert atmosphere, **EDOT-Cl** (100 mg, 0.52 mmol, 1.0 eq.) was dissolved in anhydrous DMF (5.3 mL). To this solution, NaN_3 (44 mg, 0.68 mmol, 1.3 eq.) was added and the solution was heated to 120 °C and stirred at this temperature for 24 h. After this time, the solution was cooled to room temperature and H_2O (5.0 mL) was added. The mixture was extracted with EtOAc (50 mL) and washed with H_2O (1 × 50 mL) and brine (3 × 50 mL). Combined organic fractions were dried over $MgSO_4$, filtered and concentrated *in vacuo*. The crude material was purified by column chromatography over silica gel, with the product **EDOT-N_3** eluting in 10% EtOAc in hexane as a pale yellow oil (101 mg, 99%). R_f = 0.29

^{1}H NMR (400 MHz, $CDCl_3$): δ 6.37 (dd, 2H, *J* = 16.0 Hz, *J* = 3.7 Hz), 4.34-4.29 (m, 1H), 4.20 (dd, 1H, *J* = 11.7 Hz, *J* = 2.3 Hz), 4.05 (dd, 1H, *J* = 11.7 Hz, *J* = 6.8 Hz), 3.60-3.47 (m, 2H). **^{13}C NMR** (101 MHz, $CDCl_3$): δ 141.2, 140.8, 100.4, 100.2, 72.5, 65.9, 50.6. **GCMS** (EI): *m/z* calcd. for $C_7H_7N_3O_2S$ (M^+) 197.0; found 197.0, in accordance with literature.[4]

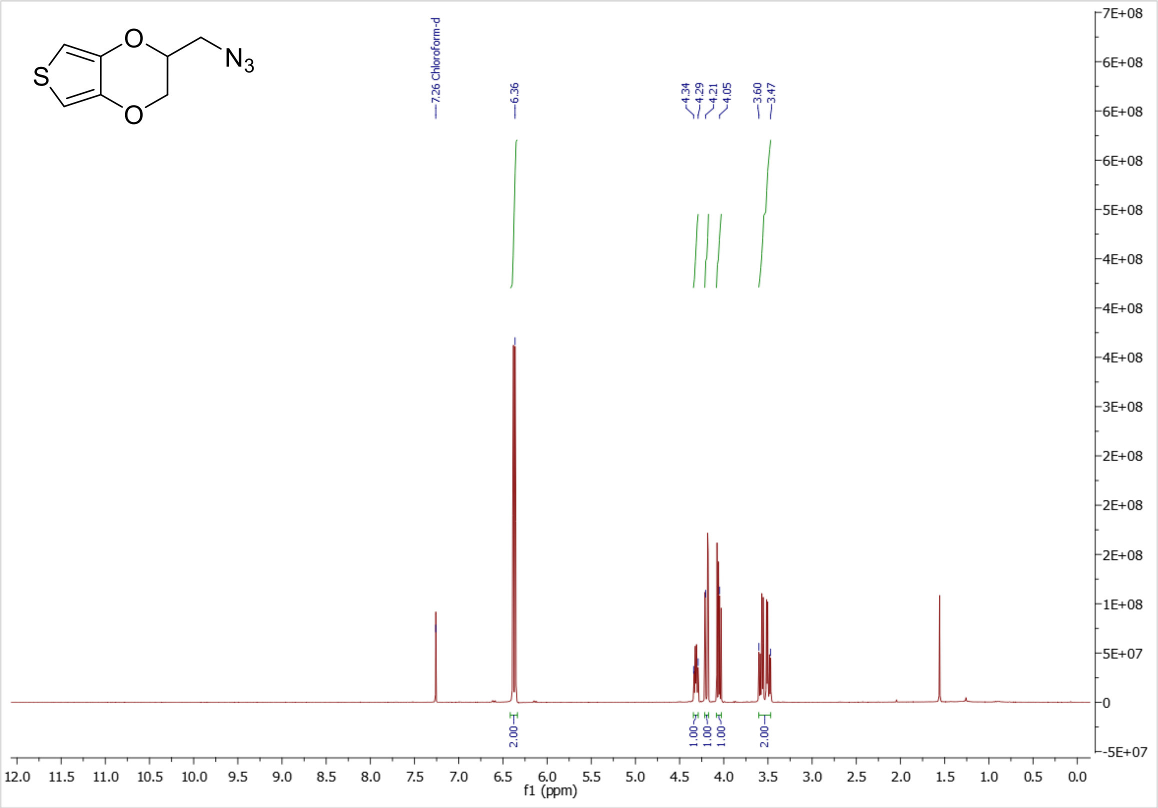

O
S
O
N3
7.26 Chloroform-d
6.36
4.34
4.29
4.21
4.05
3.60
3.47
2.00
1.00
1.00
1.00
2.00
f1 (ppm)

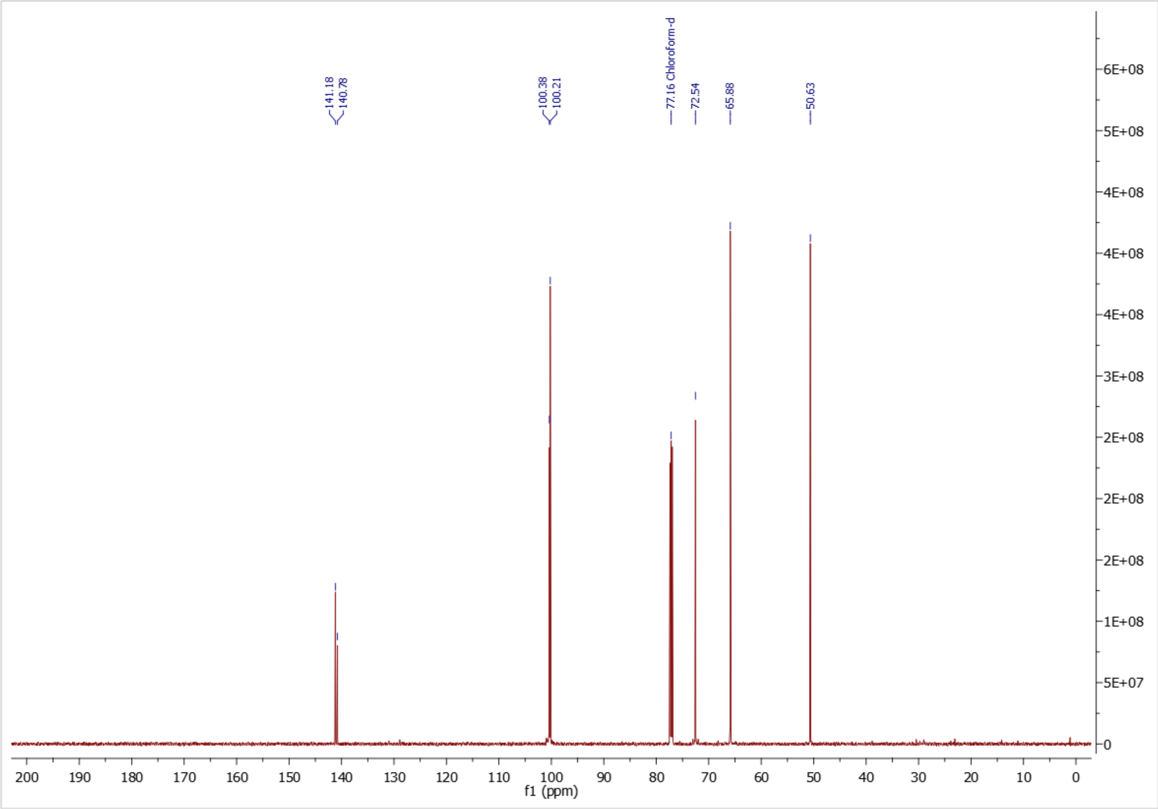

141.18
140.78
100.38
100.21
77.16 Chloroform-d
72.54
65.88
50.63
f1 (ppm)

4-Bromo-*N*-((2,3-dihydrothieno[3,4-*b*][1,4]dioxin-2-yl)methyl)benzamide **EDOT-PBr**

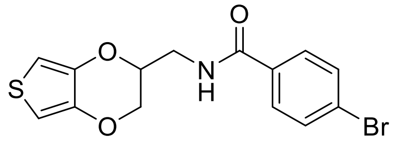

Under an atmosphere of nitrogen, 4-bromobenzoic acid (153 mg, 0.760 mmol, 1.5 eq.) was dissolved in anhydrous THF (10 mL) and DMAP (136 mg, 1.12 mmol, 2.2 eq.) was added. The mixture was cooled to 0 °C, and 1-ethyl-3-(3-dimethylaminopropyl)carbodiimide (EDC, 142 mg, 0.913 mmol, 1.8 eq.) was added. The reaction was warmed to ambient temperature and stirred for 30 min. After this time, **EDOT-N$_3$** (100 mg, 0.507 mmol, 1.0 eq.), dissolved separately in anhydrous THF (2.1 mL), was added. The mixture was cooled to 0 °C, and tributylphosphine (253 µL, 1.01 mmol, 2.0 eq.) was added dropwise. The reaction was warmed to ambient temperature and stirred for a further 3 h, then concentrated *in vacuo*. The crude material was purified by column chromatography over silica gel in a gradient of 10-30% EtOAc in hexane. The product was collected as a white powder, which was dissolved in the minimum amount of hot $CHCl_3$ and precipitated in hexane, filtered and dried to afford the pure product **EDOT-PBr** as a white solid (130 mg, 72%). R_f = 0.45 (1:1 EtOAc:hexane)

^{1}H NMR (400 MHz, $CDCl_3$): δ 7.67-7.57 (m, 4H), 6.48 (broad s, 1H), 6.36 (s, 2H), 4.41-4.36 (m, 1H), 4.28 (dd, 1H, *J* = 11.8 Hz, *J* = 2.2 Hz), 3.99 (dd, 1H, *J* = 12.0 Hz, *J* = 7.6 Hz), 3.91-3.85 (m, 1H), 3.71-3.64 (m, 1H). **^{13}C NMR** (101 MHz, $CDCl_3$): δ 167.0, 141.4, 141.2, 132.9, 132.1, 128.8, 126.7, 100.3, 100.1, 72.8, 66.4, 40.3. **HRMS:** (ES+) *m/z* calc. for $C_{14}H_{13}BrNO_3S$ (MH^+) 353.9794 (^{79}Br), 355.9774 (^{81}Br); found 353.9699 (^{79}Br), 355.9689 (^{81}Br). **mp:** 155-157 °C.

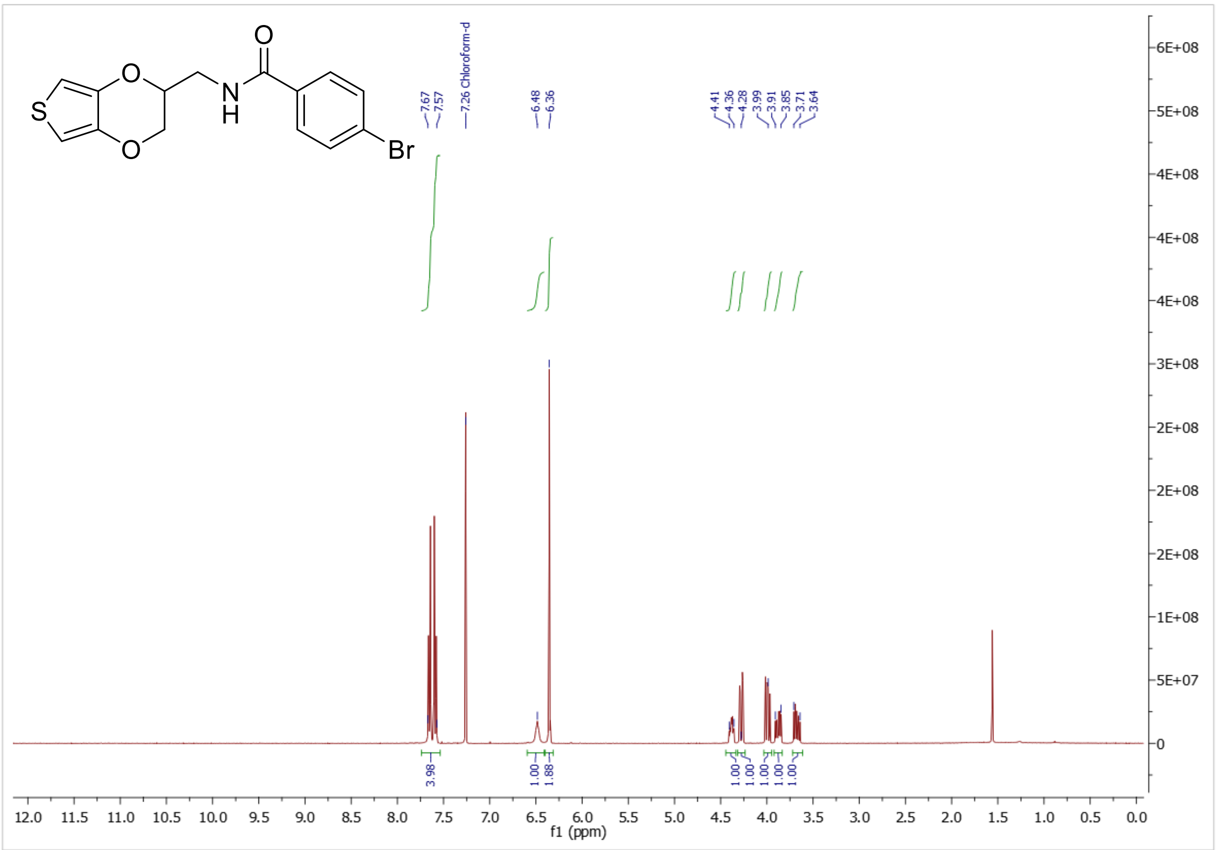
S
O
O
N
H
O
Br
7.67
7.57
7.26 Chloroform-d
6.48
6.36
4.41
4.36
4.28
3.99
3.91
3.85
3.71
3.64
3.98
1.00
1.88
1.00
1.00
1.00
1.00
1.00
f1 (ppm)

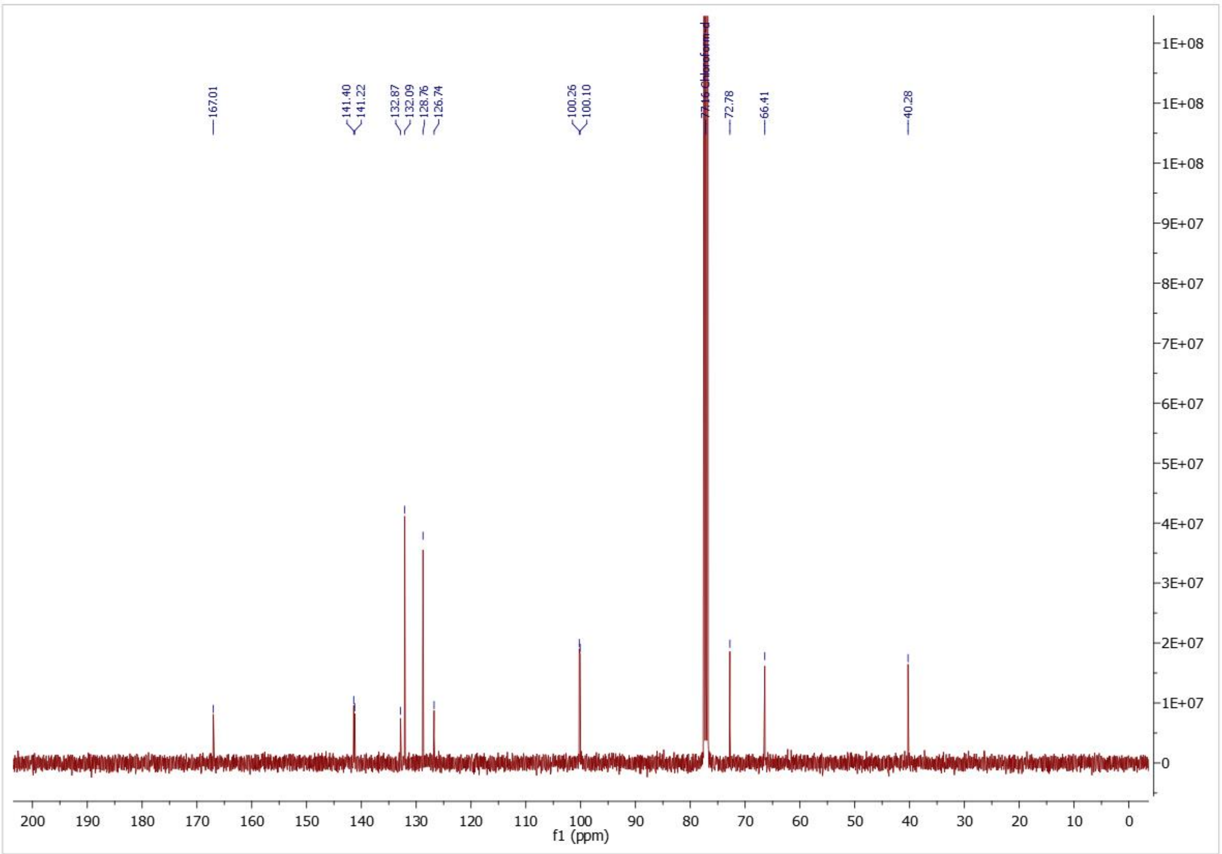
167.01
141.40
141.22
132.87
132.09
128.76
126.74
100.26
100.10
77.16 Chloroform-d
72.78
66.41
40.28
f1 (ppm)

4-(((2,3-Dihydrothieno[3,4-*b*][1,4]dioxin-2-yl)methyl)carbamoyl)phenyl)boronic acid **EDOT-PBA**

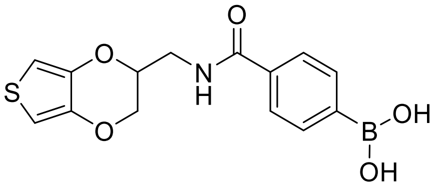

Under anhydrous conditions in a degassed 5 mL microwave vial, **EDOT-PBr** (1.79 g, 5.06 mmol, 1.0 eq.), KOAc (1.24 g, 12.7 mmol, 2.5 eq.) and chloro[(tri-tert-butylphosphine)-2-(2-aminobiphenyl)] palladium(II) (P(*t*-Bu)$_3$ Pd G2, 25.9 mg, 50.6 µmol, 1.0 mol%) were suspended in degassed MeOH (33 mL). Separately, a mixture of ethylene glycol (1.79 mL, 31.6 mmol, 6.25 eq.) and tetrahydroxydiboron (567 mg, mmol, 1.2 eq.) in degassed MeOH (17 mL) was prepared, and both mixtures were heated to 35 °C. After 10 min at this temperature, the tetrahydroxydiboron was fully dissolved, and this solution was added dropwise slowly to that of the combined solid reagents. The reaction was stirred at 35 °C for 16 h, after which it was cooled to ambient temperature, and H_2O (50 mL) was added. The suspension was filtered, and a 1:1 mixture of H_2O:MeOH (2 × 50 mL) followed by H_2O (200 mL) were passed through the grey solid. The filtrate was left to stand for 24 h, upon which the MeOH was evaporated and a white precipitate was formed, which was collected and triturated in Et_2O (100 mL). The product was filtered and dried under vacuum at 40 °C for 24 h, to give the product **EDOT-PBA** as a white solid (1.08 g, 67%).

^{1}H NMR (400 MHz, DMSO-*d*$_6$): δ 8.71 (t, 1H, *J* = 5.6 Hz), 8.17 (s, 2H), 7.84 (q, 4H, *J* = 8.4 Hz), 6.59 (s, 2H), 4.36-4.31 (m, 1H), 4.28 (dd, 1H, *J* = 11.8 Hz, *J* = 2.2 Hz), 3.97 (dd, 1H, *J* = 12.0 Hz, *J* = 7.2 Hz), 3.61-3.47 (m, 2H). **^{13}C NMR** (101 MHz, DMSO-*d*$_6$): δ 166.8, 141.3 (× 2), 135.3, 133.9, 126.2, 99.8, 99.7, 72.2, 66.1 (additional expected signal obscured by solvent residual peak). **HRMS**: (ESI) *m/z* calcd. for $C_{14}H_{15}BNO_5S$ (MH^+) 320.0759; found 320.0754. **mp:** 139-141 °C.

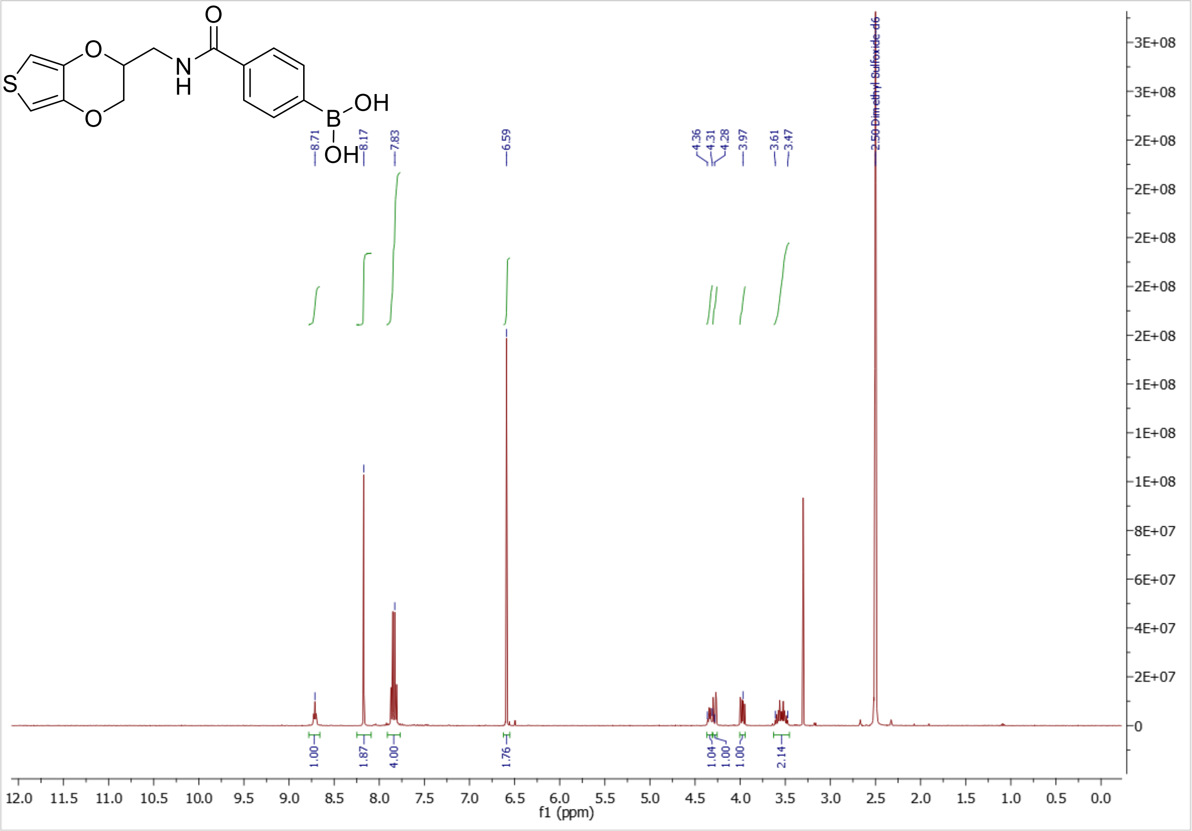

OH
B
OH
O
N
H
S
O
O
8.71
8.17
7.83
6.59
4.36
4.31
4.28
3.97
3.61
3.47
2.50 Dimethyl Sulfoxide-d6
1.00
1.87
4.00
1.76
1.04
1.00
1.00
2.14
f1 (ppm)

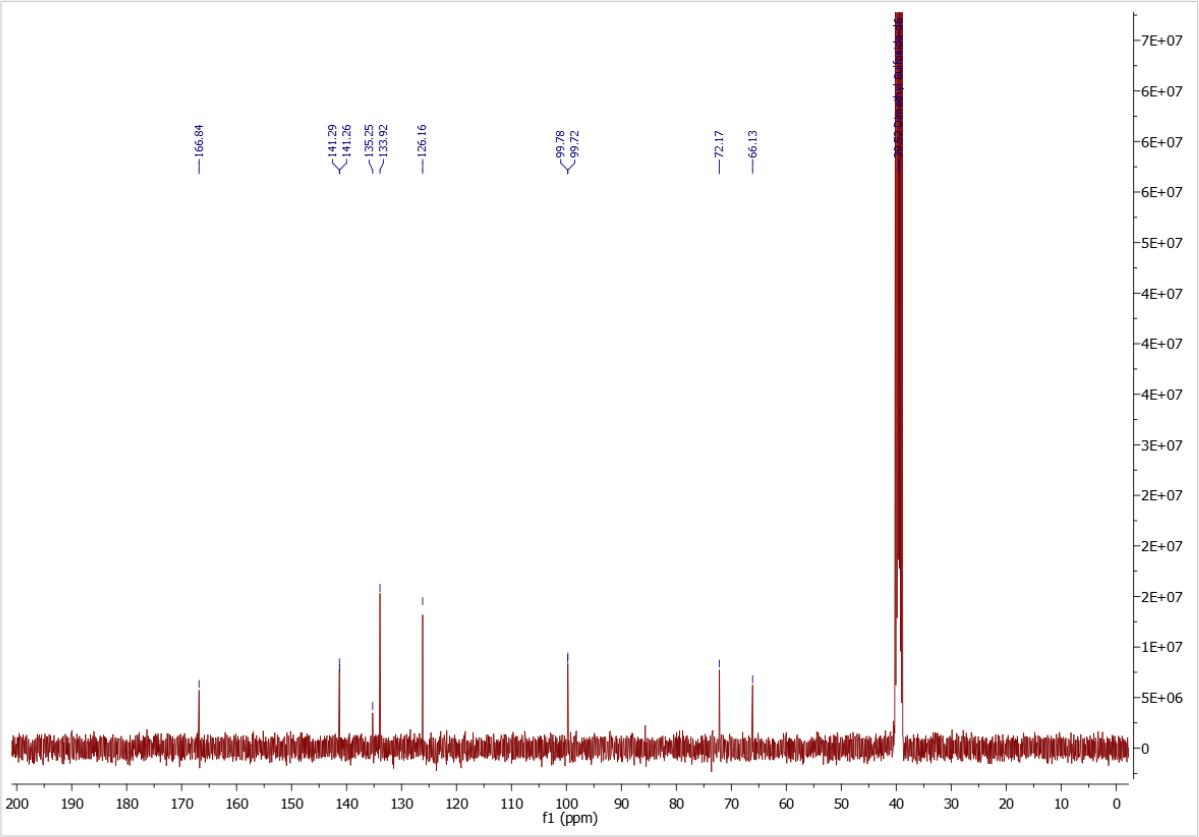

166.84
141.29
141.26
135.25
133.92
126.16
99.78
99.72
72.17
66.13
f1 (ppm)

PEDOT control for templated films

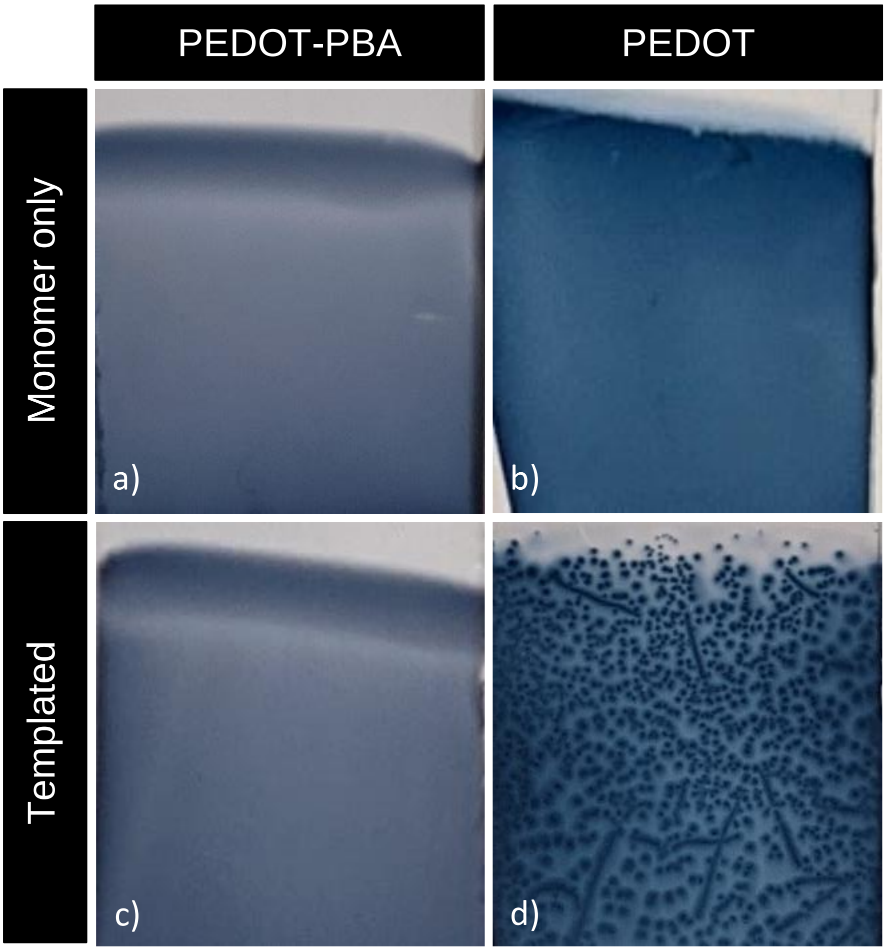

Figure S1. Photographs of polymer films of **(a,c) PEDOT-PBA** or **(b,d)** unmodified **PEDOT** polymerized by potentiostatic electrodeposition (+1.2 V, 45 sec) on ITO-glass (10 × 10 mm), from solutions of 0.1 M $TBAClO_4$ in MeCN with **(a,b)** 10 mM monomer only; **(c,d)** 10 mM monomer and 5 mM methyl α-D-glucopyranoside template.

^{1}H NMR binding study EDOT-PBA:methyl α-D-glucopyranoside

Boronic acids are known to form cyclic esters with both 1,2- and 1,3-diols;[5] thus, while various sources report boronic acids binding to glucose in either a 1:1[6] or 2:1[7,8] ratio, they do not provide consensus on which of the glucose -OH moieties are involved in this interaction, attributed to the difficulty of assigning saccharide protons using ^{1}H NMR spectroscopy due to overlapping signals in the region 3-4 ppm.[8] For this reason, our structural representations of the binding interaction between **EDOT-PBA** and methyl α-D-glucopyranoside in **Figures S2-S4** are for illustrative purposes only; however, we demonstrate that **EDOT-PBA** does bind methyl α-D-glucopyranoside in acetonitrile, with evidence for a mixture of both 2:1 and 1:1 binding occurring, detailed below.

Solutions of **EDOT-PBA** and methyl α-D-glucopyranoside alone or in the molar ratios 1:1 or 2:1 were prepared in MeCN-d_3 and analyzed by ^{1}H NMR spectroscopy**.** Examination of the resulting spectra for these mixtures identifies several features which support the evidence for monomer:template binding in the given solvent system (**Figure S2**). For **EDOT-PBA**, the doublets at 7.84 ppm representing the phenyl protons undergo a change in multiplicity from a well-resolved doublet to a multiplet, perhaps as a result of the newly asymmetric tetrahedral boron neighbour (**Figure S2 (a)-(c)**). For the sugar, the chemical shift of the doublet representing the C1 ring proton of the sugar, originally at 4.64 ppm, is shifted to 4.74 ppm, while the singlet at 3.35 ppm, representing the methoxy -CH_3 of the sugar, is shifted to 3.40 ppm (**Figure S2 (b)-(d)**) upon binding.

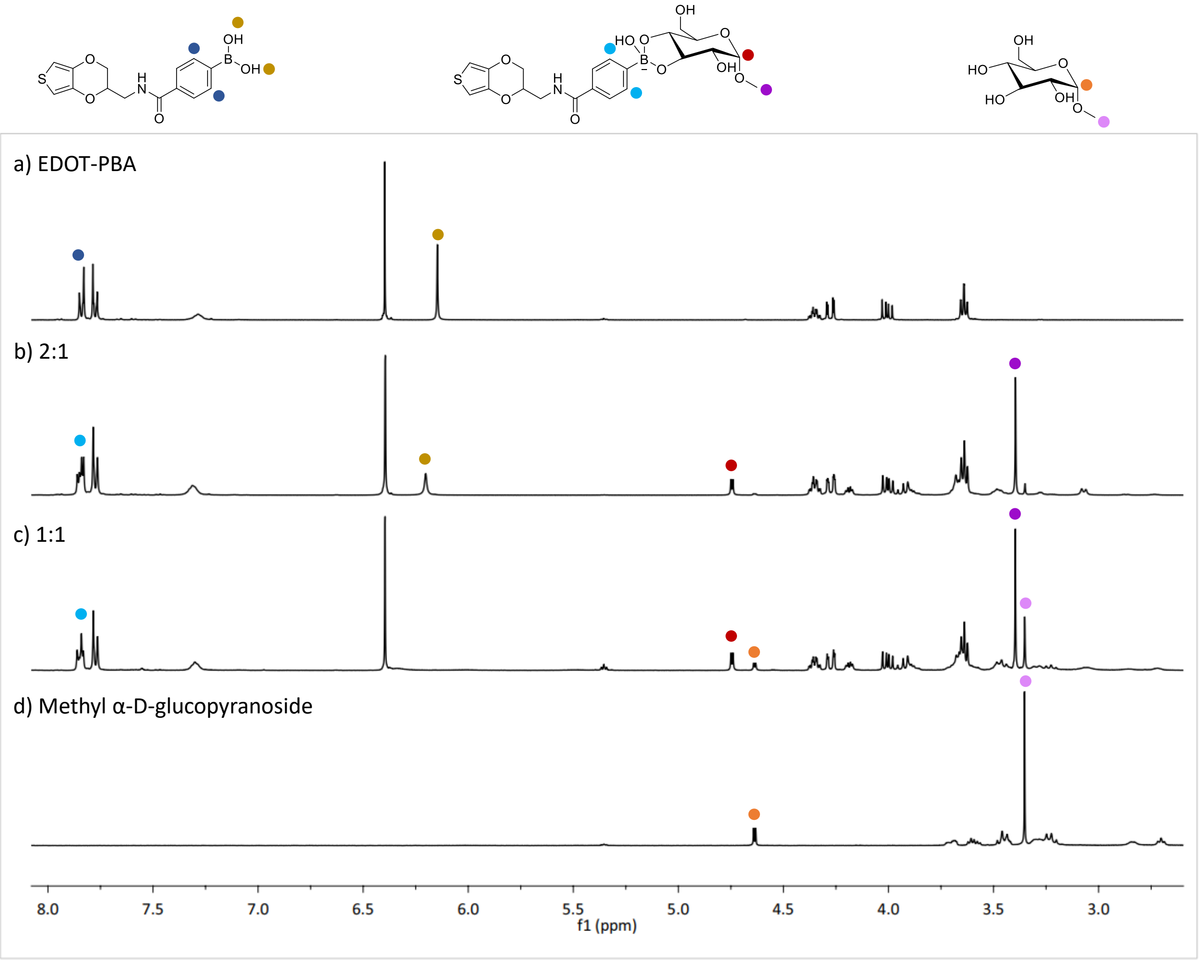

Figure S2. ^{1}H NMR spectra (400 MHz, MeCN-d_3, 126 scans) of **(a) EDOT-PBA** only; **(b)** 2:1 mixture of **EDOT-PBA** and methyl α-D-glucopyranoside; **(c)** 1:1 mixture of **EDOT-PBA** and methyl α-D-glucopyranoside; **(d)** methyl α-D-glucopyranoside only, with colours denoting the appearance of known proton environments for bound/unbound species in each mixture.

Complete 2:1 binding is ruled out by the presence of the boronic acid protons of **EDOT-PBA**, (6.15 ppm, **Figure S3 (a)**) in a 2:1 mixture of monomer:template, (**Figure S3 (b)**) and with full quenching of this signal only observed in the 1:1 mixture (**Figure S3 (c)**).

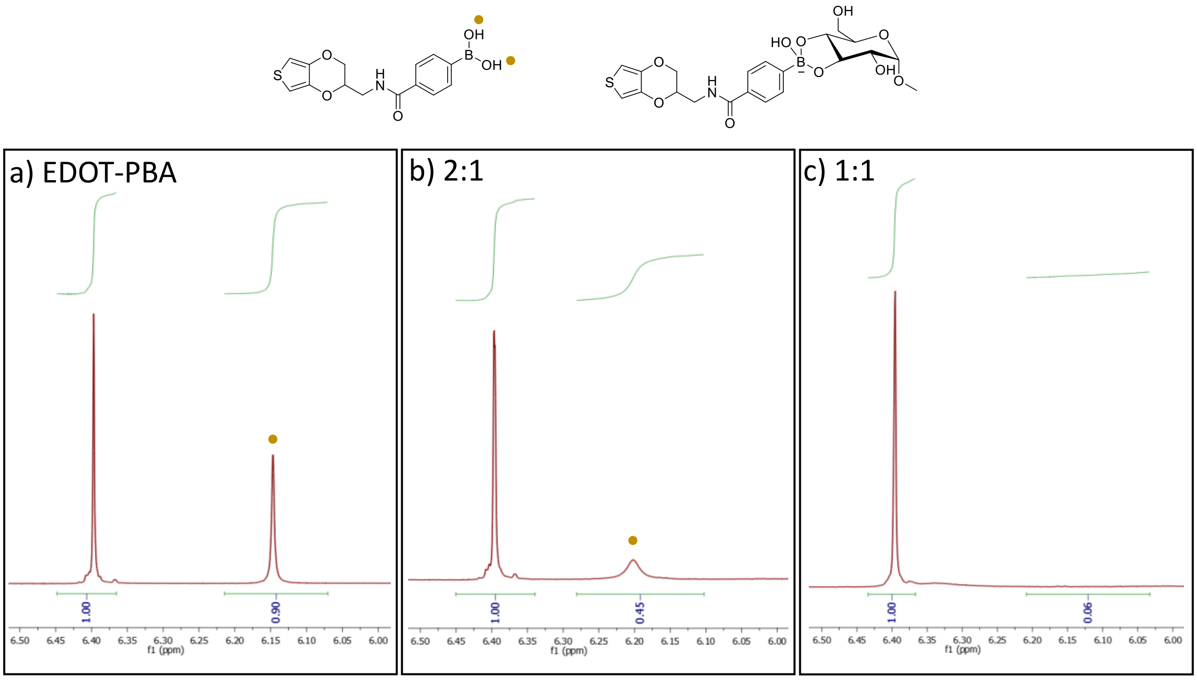

Figure S3. Comparing the integration of the -B-OH proton peak at 6.15 ppm in ^{1}H NMR spectra for **(a) EDOT-PBA**, **(b)** 2:1 mixture of **EDOT-PBA** and methyl α-D-glucopyranoside, **(c)** 1:1 mixture of **EDOT-PBA** and methyl α-D-glucopyranoside; all in MeCN-d_3. Values are normalized to the neighbouring singlet at 6.40 ppm, representing the thiophene protons of **EDOT-PBA**.

Meanwhile, entirely 1:1 binding is ruled out by the observation of unbound methyl α-D-glucopyranoside in the 1:1 mixture. (**Figure S4**).

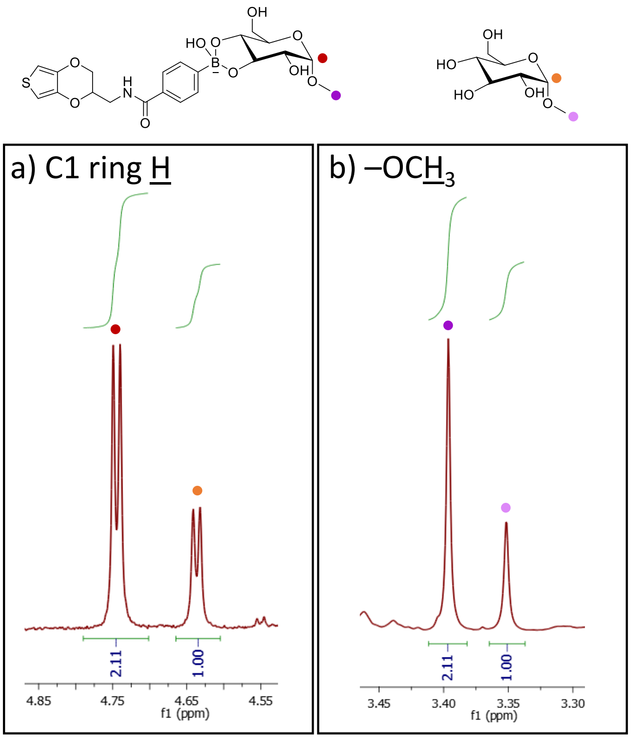

Figure S4. Comparing the integration of **(a)** the C1 ring proton in the bound (4.74 ppm) and unbound (4.64 ppm) forms and **(b)** the methoxy -OC<u>H</u>$_3$ in the bound (3.40 ppm) and unbound (3.35 ppm) forms of methyl α-D-glucopyranoside, in a 1:1 mixture of **EDOT-PBA** and methyl α-D-glucopyranoside in MeCN-d$_3$. Both environments display an approximately 2:1 ratio of bound:unbound saccharide.

While this combined evidence paints a picture of a complex system of mixed 1:1 and 2:1 binding occurring overall, we establish with confidence the novel monomer **EDOT-PBA** binding to the template methyl α-D-glucopyranoside in acetonitrile.

Spectroelectrochemistry

In this analysis, a stepwise increase in voltage was applied to NIP or MIP films on indium tin oxide (ITO)-coated glass slides in 1X PBS, while concurrently performing UV-Vis-NIR measurements. In many ways, the two systems exhibit similar behavior (**Figure S5**): discrete polaronic, then bipolaronic species formation phases up to and after 0.0 V are observed, with comparable absorption λ_{max} values of 592 nm (NIP) and 598 nm (MIP) for the neutral absorption band, and 940 nm and 956 nm respectively for the polaron; while both architectures demonstrate efficient bleaching of the neutral absorption feature mostly within a narrow voltage range of -0.3 V to 0.0 V (**Figure S6**). However, for the NIP film, a well-defined shoulder in the neutral absorption band is present at 640 nm, which is quenched at a lower potential in comparison to the main neutral peak. In the solid state, this feature can be attributed to the 0-0 vibronic transition in a H- or J-like aggregated system, which denotes a high degree of order corresponding to backbone planarity in the polymer.[9,10] This feature is far less pronounced for the MIP film, indicating a more amorphous structure for this film architecture.

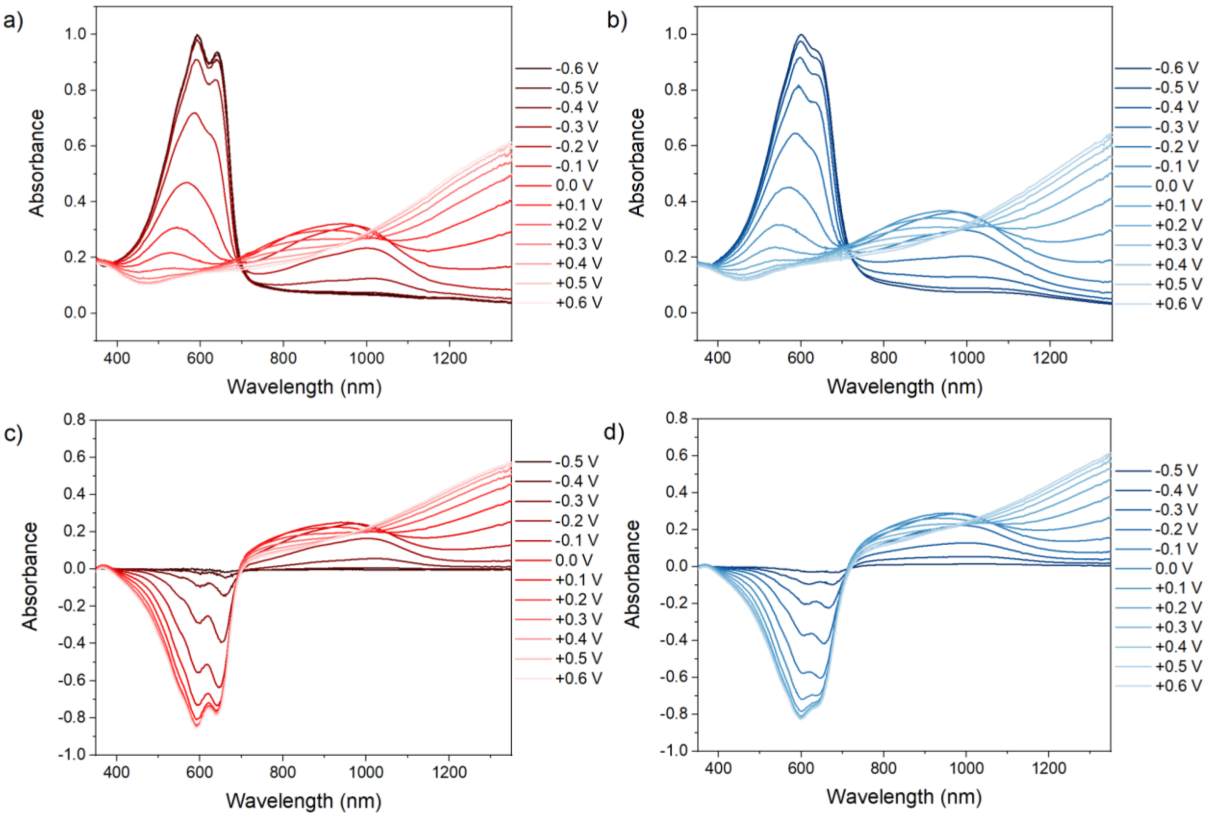

Figure S5. Spectroelectrochemical analysis from -0.6 – +0.6 V of **PEDOT-PBA** thin films, polymerized on ITO-glass by potentiostatic electrodeposition (+1.2 V, 45 s) in 1X PBS (pH 7.4) **(a)** NIP and **(b)** MIP; plotted as difference *vs*. neutral polymer (at -0.6 V) for **(c)** NIP and **(d)** MIP.

Bleaching of neutral absorption band

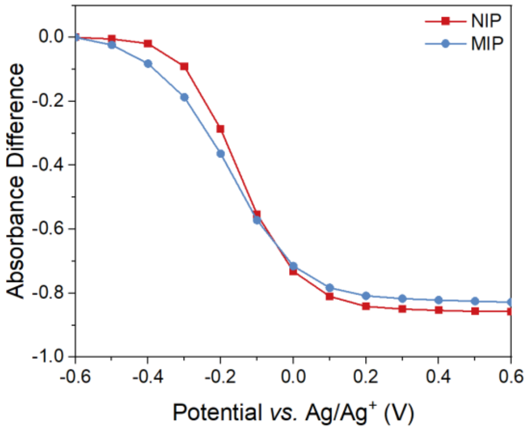

Figure S6. Bleaching of neutral absorption band (absorbance difference at λ_{max} for increasing potentials *vs*. neutral polymer at -0.6 V) for NIP (recorded at 592 nm) and MIP (recorded at 598 nm).

EIS measurement of NIP and MIP electrodes

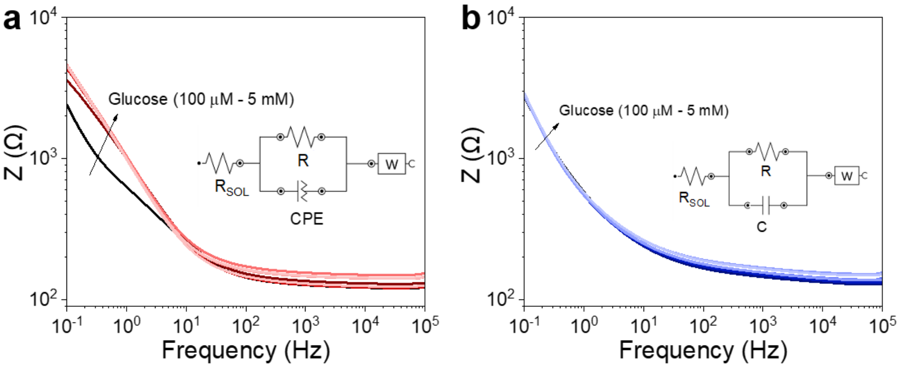

Figure S7. Electrochemical impedance spectra (Bode plots) of **(a)** NIP and **(b)** MIP polymer films before and after interactions with glucose at different concentrations. The circuits shown in the insets were used to fit the spectra. R_{SOL} is solution resistance. R, CPE, and C are the resistance, constant phase element, and capacitance of the electrode material, while W is the Warburg element.

Table S1. Parameters obtained from fitting the EIS spectra

Parameters	NIP			MIP		
	**0 M**	**100 µM**	**5 mM**	**0 M**	**100 µM**	**5 mM**
R (kΩ)	-5.07	12.4	20.7	-12.4	-12	-10.4
CPE ($\mu S \cdot s^N$)	1540	305	292	-	-	-
C (µF)	-	-	-	963	927	913
W ($mS \cdot \sqrt{s}$)	0.83	1.1	4.95	1.07	1.12	1.17
X^2 values	0.232	0.060	0.040	0.064	0.104	0.137

MIP-gated OECT characteristics

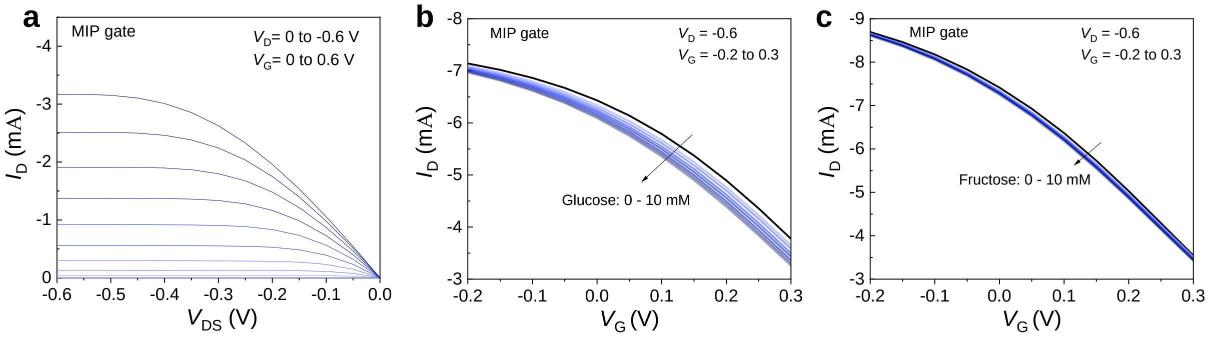

Figure S8. The OECT biosensor characteristics gated with the MIP electrodes. **(a)** The output characteristics of the OECT with the MIP electrode as the gate in PBS. Transfer characteristics of the OECT channel gated with the MIP electrodes exposed to **(b)** glucose and **(c)** fructose solutions of varying concentrations.

Statistical analysis and Limit of Detection (LOD)

Table S2. Statistical data for the calibration plots of glucose detection using NIP and MIP gate sensors

Sensor	Concentration range	R^2	Linear equation	LOD
NIP	10 µM – 100 µM	0.999	y = -0.174 log(x) – 0.98	28.2 µM*
	100 µM – 10 mM	0.977	y = -0.055 log(x) – 0.504	
MIP	10 µM – 10 mM	0.992	y = -0.032 log(x) – 0.197	22.3 µM

*The LOD for the NIP is calculated using the linear equation for the lower concentration range.

Table S3. Comparison of LOD values for non-enzymatic electrochemical PBA-based glucose sensors

Sensor	LOD	Reference
Aliphatic MIP-coated gate FET	3 µM	11
**PEDOT-PBA** MIP OECT	22.3 µM	*This work*
**PEDOT-PBA** NIP OECT	28.2 µM	*This work*
EIS of electropolymerized PEDOT-PBA (ether/amide linked alternative design)	50 µM	12
Hybrid PEDOT:PSS/PBA hydrogel amperometric electrode	61 µM	13
EIS of anthracene-diboronic acid/ $Fe(CN)_6^{3-/4-}$ immobilized electrode	21.5 mg/dL (≈1.2 mM)	14
PEDOT:PSS/PBA double network hydrogel OECT	2 mM	15
PBA-functionalized carbon nanotube FET	5 mM	16

LOD is determined using the formula LOD = 3.3 × (σ/S), where σ represents the standard deviation of the y-intercept and S is the slope of the calibration curve.

Specificity Test of MIP-gated OECT Sensors

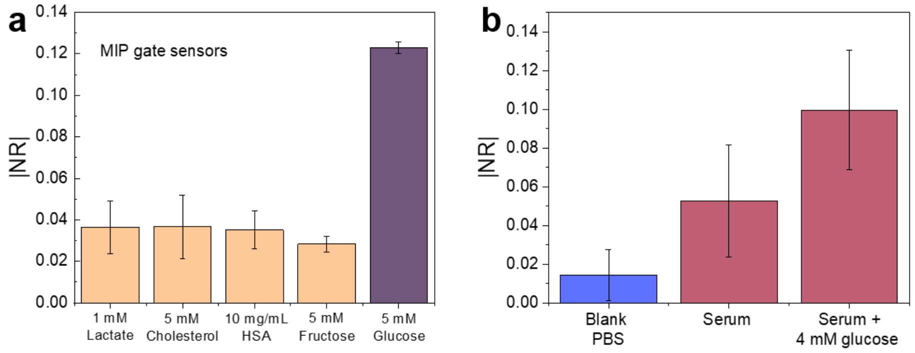

Figure S9. The specificity of MIP gated OECT biosensors. (a) Response to common interferents or abundant molecules in human serum samples at their physiological concentrations. (b) Response to PBS and commercial serum with and without the spiked additional glucose molecules.

Sensitivity Test of MIP-gated OECT Sensors

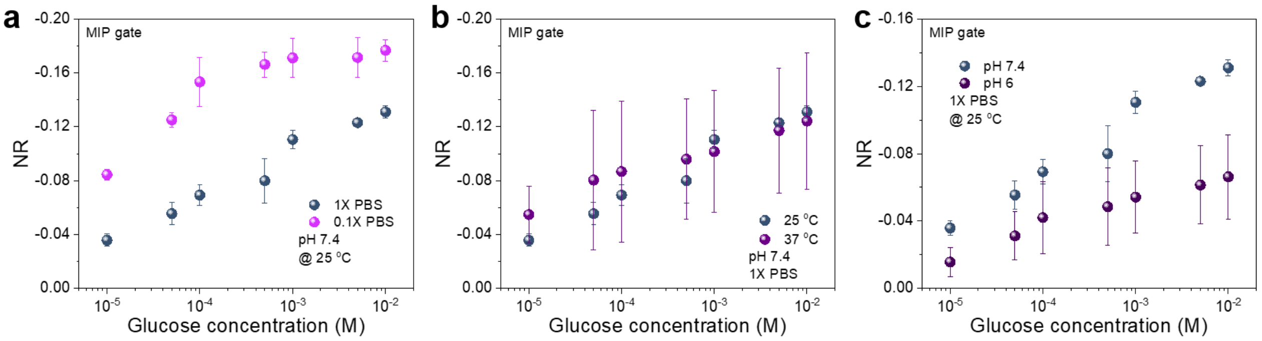

Figure S10. The performance of MIP gated OECT sensors after incubation with glucose spiked in PBS solution with different: **(a)** ionic strength, **(b)** temperature, and **(c)** pH.

References

1 C. J. Kousseff, F. E. Taifakou, W. G. Neal, M. Palma and C. B. Nielsen, *J. Polym. Sci.*, 2022, **60**, 504–516.

2 J. L. Segura, R. Gómez, E. Reinold and P. Bäuerle, *Org. Lett.*, 2005, **7**, 2345–2348.

3 S. Zhang, J. Xu, B. Lu, L. Qin, L. Zhang, S. Zhen and D. Mo, *J. Polym. Sci. A – Polym. Chem.*, 2014, **52**, 1989–1999.

4 D. Hu, B. Lu, X. Duan, J. Xu, L. Zhang, K. Zhang, S. Zhang and S. Zhen, *RSC Adv.*, 2014, **4**, 35597–35608.

5 R. Nishiyabu, Y. Kubo, T. D. James and J. S. Fossey, *Chem. Commun.*, 2011, **47**, 1124–1150.

6 H. Çiftçi, U. Tamer, M. Ş. Teker and N. Ö. Pekmez, *Electrochim. Acta*, 2013, **90**, 358–365.

7 H.-C. Wang, H. Zhou, B. Chen, P. M. Mendes, J. S. Fossey, T. D. James and Y.-T. Long, *Analyst*, 2013, **138**, 7146–7151.

8 Y. Shiomi, M. Saisho, K. Tsukagoshi and S. Shinkai, *J. Chem. Soc. Perkin 1*, 1993, 2111–2117.

9 F. C. Spano, *Acc. Chem. Res.*, 2010, **43**, 429–439.

10 P. A. Finn, I. E. Jacobs, J. Armitage, R. Wu, B. D. Paulsen, M. Freeley, M. Palma, J. Rivnay, H. Sirringhaus and C. B. Nielsen, *J. Mater. Chem. C*, 2020, **8**, 16216–16223.

11 T. Kajisa and T. Sakata, *ACS Appl. Mater. Interfaces*, 2018, **10**, 34983–34990.

12 P.-C. Huang, M.-Y. Shen, H. Yu, S.-C. Wei and S.-C. Luo, *ACS Appl. Bio. Mater.*, 2018, **1**, 160–167.

13 S. Wustoni, A. Savva, R. Sun, E. Bihar and S. Inal, *Adv. Mater. Interfaces*, 2019, **6**, 1800928.

14 J. H. Kim, H. Choi, C. S. Park, H. S. Yim, D. Kim, S. Lee and Y. Lee, *Biosensors*, 2023, **248,** 2.

15 A. C. Tseng and T. Sakata, *ACS Appl. Mater. Interfaces*, 2022, **14**, 24729–24740.

16 A. Vlandas, T. Kurkina, A. Ahmad, K. Kern and K. Balasubramanian, *Anal. Chem.*, 2010, **82**, 6090–6097.
